# Fully Reversible and Super‐Fast Photo‐Induced Morphological Transformation of Nanofilms for High‐Performance UV Detection and Light‐Driven Actuators

**DOI:** 10.1002/advs.202307165

**Published:** 2024-01-15

**Authors:** Xiangquan Liu, Jiahui Hu, Jinglun Yang, Lingya Peng, Jiaqi Tang, Xiaohui Wang, Rongrong Huang, Jianfei Liu, Kaiqiang Liu, Tingyi Wang, Xiaoyan Liu, Liping Ding, Yu Fang

**Affiliations:** ^1^ Key Laboratory of Applied Surface and Colloid Chemistry Ministry of Education School of Chemistry and Chemical Engineering Shaanxi Normal University Xi'an 710119 China; ^2^ Department of Materials Science and Engineering City University of Hong Kong Hong Kong SAR 999077 China; ^3^ Xi'an Rare Matel Materials Institute Co. Ltd Xi'an 710016 China; ^4^ Northwest Institute for Nonferrous Metal Research Xi'an 710016 China

**Keywords:** *cis–trans* isomerization, dynamic condensation, nanofilms, photo‐induced motion, UV detection

## Abstract

Flexible and highly ultraviolet (UV) sensitive materials garner considerable attention in wearable devices, adaptive sensors, and light‐driven actuators. Herein, a type of nanofilms with unprecedented fully reversible UV responsiveness are successfully constructed. Building upon this discovery, a new system for ultra‐fast, sensitive, and reliable UV detection is developed. The system operates by monitoring the displacement of photoinduced macroscopic motions of the nanofilms based composite membranes. The system exhibits exceptional responsiveness to UV light at 375 nm, achieving remarkable response and recovery times of < 0.3 s. Furthermore, it boasts a wide detection range from 2.85 µW cm^−2^ to 8.30 mW cm^−2^, along with robust durability. Qualitative UV sensing is accomplished by observing the shape changes of the composite membranes. Moreover, the composite membrane can serve as sunlight‐responsive actuators for artificial flowers and smart switches in practical scenarios. The photo‐induced motion is ascribed to the *cis–trans* isomerization of the acylhydrazone bonds, and the rapid and fully reversible shape transformation is supposed to be a synergistic result of the instability of the *cis‐*isomers acylhydrazone bonds and the rebounding property of the networked nanofilms. These findings present a novel strategy for both quantitative and qualitative UV detection.

## Graphical abstract

A rapid, sensitive, and robust UV detector is developed via monitoring the displacement of the photo‐induced macroscopic motions of some specially designed nanofilms‐based composite membranes. The membranes are also used for the fabrication of light‐driven actuators, resulting in power‐free UV sensing, LED controlling, and smart windows for UV screening.

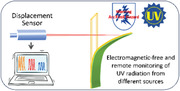

## 1 Introduction

UV light is widely utilized in both military and civilian applications.^[^ ^1^, ^2^ ^]^ For instance, shortwave UV light can serve as a transmission medium and assist in missile tracking.^[^ ^3^, ^4^ ^]^ In daily life, UV light aids the human body in absorbing calcium, though excessive exposure can lead to skin damage and even skin cancer.^[^ ^5^, ^6^ ^]^ Additionally, UV light was applied in photochemical synthesis, remote control, and medical treatments.^[^ ^1^, ^7^, ^8^ ^]^ In recent decades, significant advancements have been made in the development of UV detection based on inorganic materials, such as semiconductors and halide perovskites.^[^ ^9^, ^10^, ^11^ ^]^ In fact, with the increasing demands and expectations of wearable devices, adaptable imaging sensors, and remote monitors, the development of flexible and power‐free visualized UV sensing materials is urgently required.^[^ ^3^, ^10^, ^11^ ^]^ However, the rigid inorganic semiconductors and halide perovskites have limited flexibility and difficulties in machining, which make them be not suitable to develop flexible products.^[^ ^12^, ^13^, ^14^, ^15^, ^16^, ^17^, ^18^, ^19^, ^20^, ^21^, ^22^ ^]^ To address these challenges, flexible UV‐sensitive films were developed through the physical doping of photo‐responsive semiconducting micro‐/nanoparticles into polymeric membranes.^[^ ^23^, ^24^, ^25^ ^]^ However, the incompatibility between these two components could result in delamination or fracture, influencing the sensing performance.^[^ ^26^, ^27^ ^]^ To solve the problems, a few fully flexible and photo‐responsive materials, such as ionic liquid‐containing liquid crystalline polymer and polyurethane composite fabrics, has been developed to construct novel UV detectors.^[^ ^28^, ^29^, ^30^ ^]^ Yet, these systems still fall short of meeting expectations due to limited sensitivity, slow response time, and the potential for ionic liquid leakage.^[^ ^30^ ^]^

Recently, photo‐deformable materials have garnered considerable attention across various areas, such as energy harvesting,^[^ ^31^, ^32^ ^]^ soft robotics,^[^ ^33^, ^34^ ^]^ artificial muscles,^[^ ^35^ ^]^ and switchable devices.^[^ ^36^ ^]^ Commonly employed photo‐responsive compounds include azobenzene,^[^ ^37^ ^]^ anthracene,^[^ ^38^ ^]^ and hydrazone.^[^ ^39^, ^40^, ^41^, ^42^ ^]^ However, these materials often exhibit slow response and necessitate an extra stimulus for reversed conversion,^[^ ^35^, ^40^, ^43^, ^44^ ^]^ hindering the realization of UV sensors and self‐recovering actuators using these materials.

**Figure 1:**
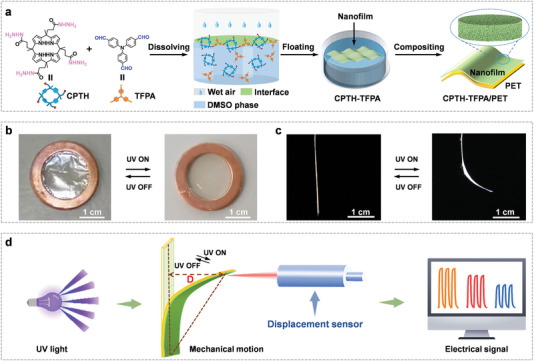
Schematic representation of the preparation of a unique nanofilm and its application in UV detection. a) Molecular structure of CPTH and TFPA, and schematic representation of the preparation process of the CPTH‐TFPA/PET membranes. b) UV‐responsive behaviors of the CPTH‐TFPA nanofilm, where the nanofilm was loaded on a copper ring and the wavelength of UV light was 375 nm. c) UV‐responsive bending behaviors of the CPTH‐TFPA/PET membrane (side view). d) Schematic diagram of the UV detection system based on the CPTH‐TFPA/PET membrane.

In this study, flexible, self‐standing, and robust nanofilms were prepared using a tetrahydrazide derivative of calix[4]pyrrole (CPTH) and tris(4‐formylphenyl)amine (TFPA) through interfacial confined dynamic condensation (**Figure** **1**a). Remarkably, a representative CPTH‐TFPA nanofilm exhibited a rapid and reversible change in surface morphology upon exposure to UV light (Figure 1b). Additionally, by laminating the nanofilm onto a commercially available thin PET membrane, a composite membrane with fully reversible shape transformation was observed (Figure 1c). Taking advantage of the photo‐responsive property of the composite membrane, a novel UV detection strategy was developed wherein the CPTH‐TFPA/PET composite membrane acted as an actuator capable of converting UV illuminance into macroscopic motion. The motion of the membrane was recorded using a displacement sensor, enabling digital UV detection (Figure 1d; Figure S5, Supporting Information). Fortunately, the membrane‐based system exhibited super‐fast response and recovery, high detection sensitivity, a wide illuminance detection range, and excellent performance stability.^[^ ^27^, ^30^ ^]^ Furthermore, the photo‐induced reversible morphological change of the nanofilm‐based composite membranes facilitated power‐free visualized sensing of UV light and smart switches via UV stimulus, thus opening up possibilities for various applications.

## 2 Results and Discussion

### 2.1 Fabrication of CPTH‐TFPA Nanofilms

First, the CPTH monomer (Scheme S1 and Figures S1–S3, Supporting Information) was synthesized.^[^ ^45^ ^]^ CPTH and TFPA dissolve in DMSO and can self‐assemble at the humid air‐DMSO interface. This enables the formation of CPTH‐TFPA nanofilms through interfacial confined dynamic condensation at room temperature (25 °C) and medium humidity (≈50%) (Figure 1a), which have been optimized (Figure S4, Supporting Information).^[^ ^46^, ^47^, ^48^ ^]^ The CPTH‐TOFB and CPTH‐ETBA nanofilms were prepared in a similar manner, using different reactive monomers (Scheme S2, Supporting Information).

**Figure 2:**
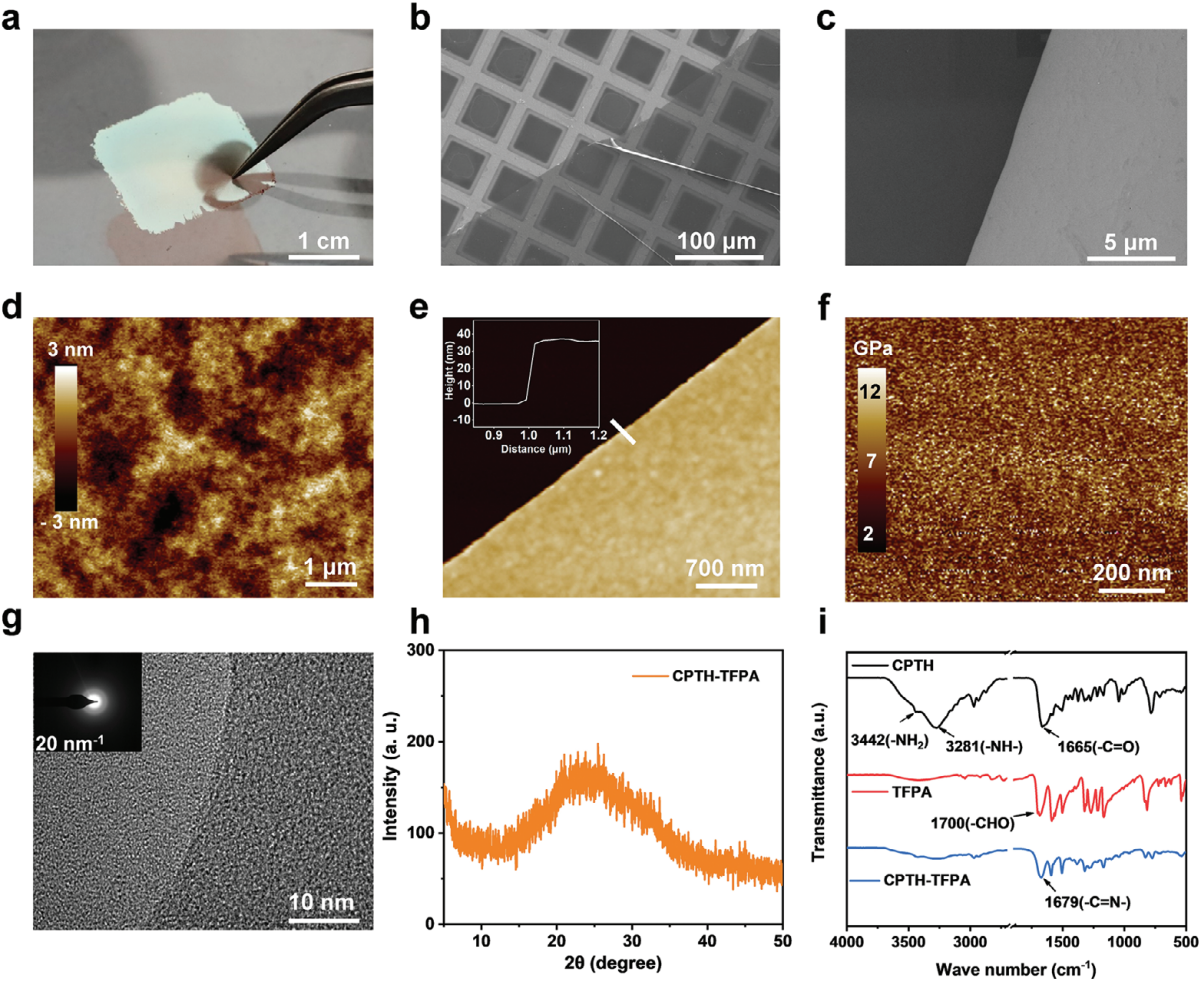
Photograph and characterization of the CPTH‐TFPA nanofilms. a) The CPTH‐TFPA nanofilm floating on the water surface. b,c) SEM images of the nanofilm. d,e) AFM images of the surface and boundary of the CPTH‐TFPA nanofilm supported on a silicon wafer, where the inset in e is the corresponding AFM height profile. f) The Young's modules of CPTH‐TFPA nanofilm. g) A HRTEM image of the CPTH‐TFPA nanofilm (right half part), where the inset is the electron diffraction pattern generated during the measurement. h) XRD trace of the CPTH‐TFPA nanofilm. i) FTIR spectra of the building blocks and the obtained CPTH‐TFPA nanofilm.

**Figure** **2**a shows that the prepared CPTH‐TFPA nanofilm can float on the water surface and is resistant to pressure from thin‐tipped tweezers. The contact angles of the two sides of the CPTH‐TFPA nanofilms are 73° (air side) and 46° (DMSO side), respectively (Figure S6, Supporting Information). This difference in contact angles could be attributed to the orientation of the amphipathic CPTH and TFPA molecules. SEM images confirm that the surface of the nanofilms are smooth, uniform, and defect‐free (Figure 2b,c). The other two nanofilms (CPTH‐ETBA and CPTH‐TOFB) exhibit similar structures and properties (Figure S7, Supporting Information). The observed wrinkles in the nanofilm (Figure 2b) indicate its flexibility. AFM measurements reveal that the nanofilm, prepared at a precursor concentration of 0.5 wt%, has a surface roughness of 0.76 nm (Figure 2d) and a thickness of 35 nm (Figure 2e). Furthermore, the thickness of the nanofilms can be controlled by adjusting the concentration of the precursor solution, with a range of at least 35 to 100 nm (Figure 2e; Figure S8, Supporting Information). The mechanical properties of the nanofilm were evaluated using AFM in the Fast Force Mapping mode, with a determined Young's modulus of ≈9.3 GPa (Figure 2f). HRTEM and XRD measurements indicate the amorphous nature of the prepared nanofilms (Figure 2g,h), likely due to the flexibility of the acylhydrazone bond‐featured crosslinking structures and fast reaction rate. Infrared spectroscopy studies confirm the presence of the expected acylhydrazone bonds through the appearance of characteristic absorption peaks of C═N at 1679 cm^−1^ and decreased aldehyde stretching vibration peaks at 1700 cm^−1^ (Figure 2i).^[^ ^43^, ^47^ ^]^ XPS tests reveal new peaks of C═N (286 eV) and C═N (401 eV) in the C 1s and N 1s regions, respectively, further confirming the conversion of aldehyde structures to the anticipated acylhydrazone bonds (Figure S9, Supporting Information).^[^ ^43^, ^47^ ^]^

### 2.2 Photo‐Induced Motions of Nanofilms and Composite Membranes

The changes in surface morphology of nanofilms and shape transformations of laminated composite membranes upon UV irradiation was investigated. In the case of the nanofilm supported on a copper ring, its morphology changed instantaneously from a very plicated state to a smooth one upon UV irradiation (Figure 1b). This change was reversed when the UV light was removed (Video S1, Supporting Information). Interestingly, the nanofilm‐based composite membrane (CPTH‐TFPA/PET) exhibited fully reversible and rapid macroscopic motions upon UV light exposure and subsequent removal (Figure 1c; Video S2, Supporting Information), which is a rarely reported phenomenon as most photo‐deformable materials require another stimulus to return to their initial state.^[^ ^49^, ^50^ ^]^ Based on this discovery and the strong adhesion of the nanofilm to the substrate membrane (part 5, Figure S10, Supporting Information), a displacement sensor was used to quantify the degree of bending, with the goal of developing a new type of UV detector (Figure 1d). To optimize the detector's performance, the effect of nanofilm thickness on its response was examined when supported on the PET membrane. It was observed that the composite membrane with six layers of nanofilm showed the highest response under a given UV irradiation (Figure S11, Supporting Information). This result implies that an increased thickness of the nanofilm allows for better utilization of the irradiated UV light, as it increases the number of response sites. However, further increasing the nanofilm thickness may exceed the range of UV light irradiation and lead to increased rigidity of the membrane, which is unfavorable to bending. Moreover, there is no difference in the UV responsiveness of the nanofilm at the temperature range from 10 to 80 °C. Notably, the composite membranes showed a humidity responsiveness (Figure S12, Supporting Information) that would be interesting for future investigation.

### 2.3 UV Detection Performance of Nanofilm Based Composite Membrane

The prepared CPTH‐TFPA nanofilms exhibited a broad absorption peak at 375 nm (Figure S13a, Supporting Information). Similarly, the CPTH‐TOFB and CPTH‐ETBA nanofilms displayed absorptions at 275 and 310 nm, respectively (Figure S13a, Supporting Information). These absorption bands are primarily determined by the aldehyde monomers used (Figure S13b, Supporting Information), suggesting that the wavelength for UV detection can be easily adjusted by altering the building blocks of the nanofilms. This expands the potential applications of these photo‐deformable nanofilms. For instance, by changing the composite membrane employed, the UV detector could be utilized to monitor UVA (400–320 nm) that leads to skin aging, or UVB (320‐280 nm) that causes skin inflammation.^[^ ^51^ ^]^

**Figure 3:**
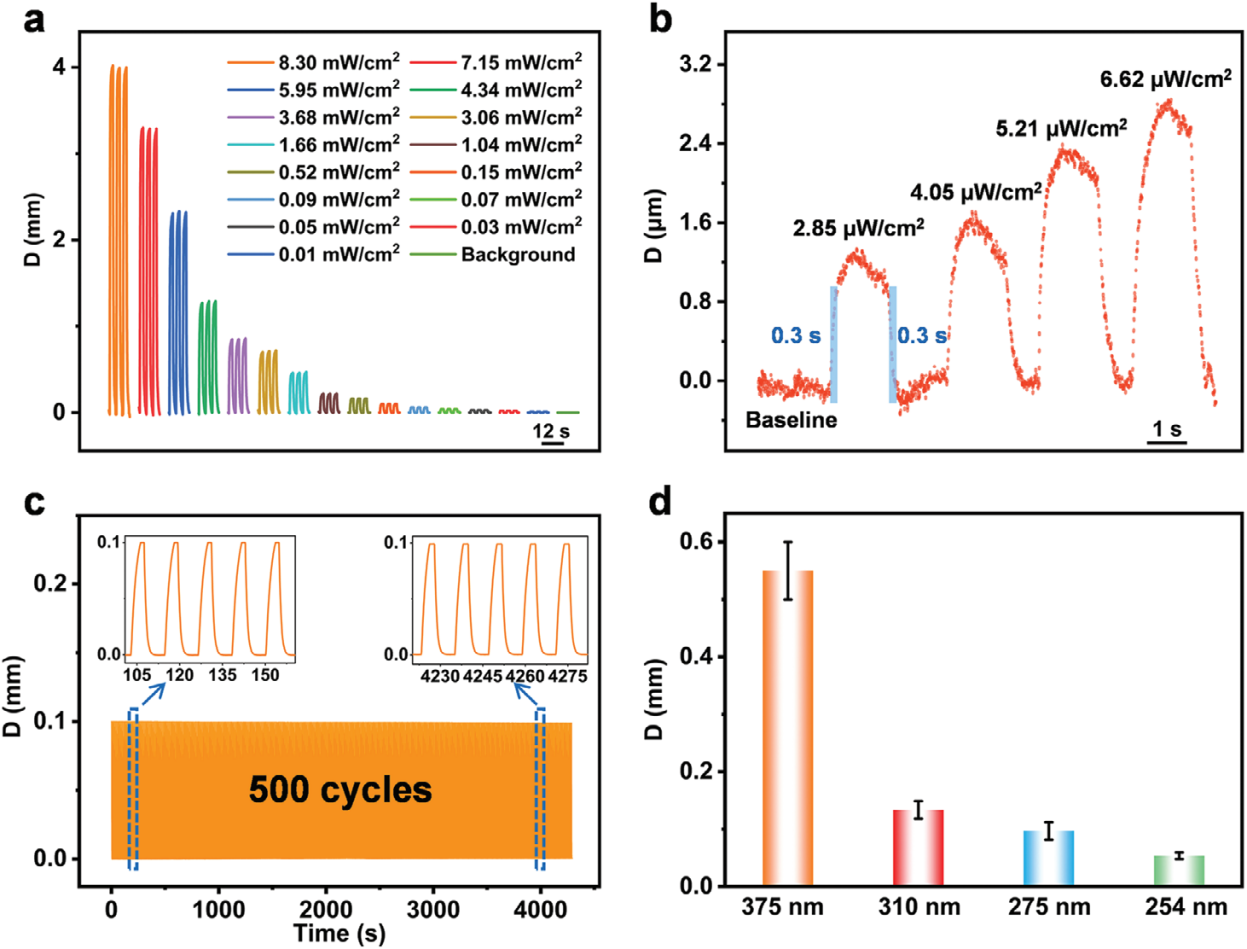
Sensing performance of the CPTH‐TFPA/PET composite membrane for UV light. a,b) The response of the membrane to 375 nm UV light at different illuminances, where the parts highlighted in blue is a detailed analysis of the response and recovery curves of nanofilm to the light at 2.85 µW cm^−2^. c) The responses of the membrane upon 500 consecutive UV (375 nm, 0.15 mW cm^−2^) on/off illuminations, where the insets are the amplified five on/off responses extracted, respectively, from the front‐end and back‐end of the whole response traces. d) Responses of the membrane to UV light of different wavelengths, where the UV illuminance is 0.1 mW cm^−2^.

The displacement distances of the CPTH‐TFPA/PET composite membrane‐based UV detector in response to UV irradiation at 375 nm with varying intensities were systematically examined. As shown in **Figure** **3**a, the composite membrane exhibited macroscopic motion upon exposure to UV light (Video S3, Supporting Information), and the displacement signal increased with increasing power of the UV light. Quantitative analysis revealed that detection could range from 2.85 µW cm^−2^ to 8.30 mW cm^−2^ (Figure 3a), with a linear range < 10 µW cm^−2^ and a correlation coefficient of 0.99 (Figure S14, Supporting Information). The response and recovery times were < 0.3 s for 2.85 µW cm^−2^ UV illuminance (Figure 3b). This rapid response time and high sensitivity can be attributed to the large number of photo‐responsive acylhydrazone bonds in the nanofilm, as well as the high Young's modulus of the nanofilm.^[^ ^27^ ^]^ Furthermore, the response amplitude and speed of the last five testing cycles from the 500 consecutive tests were comparable to the initial five cycles under irradiation of 0.15 mW cm^−2^ UV light, indicating the stability of the developed UV detector (Figure 3c). Additionally, one time a day for 16 days and about six month storage UV‐responsiveness tests showed the robust durability of the nanofilm (Figures S15 and S16, Supporting Information). The maximum UV‐vis absorption at 375 nm of the CPTH‐TFPA nanofilm allows for a selective response to UV light at this wavelength (Figure 3d). Additional investigations demonstrated that the CPTH‐TOFB and CPTH‐ETBA nanofilm‐based UV detectors can effectively detect UV light at 254 nm (Figures S17 and S18, Supporting Information) and 310 nm (Figures S19 and S20, Supporting Information), respectively. Similarly, these two detectors also exhibit fast, sensitive, and stable performances.

### 2.4 Visualized Sensing of UV light

**Figure 4:**
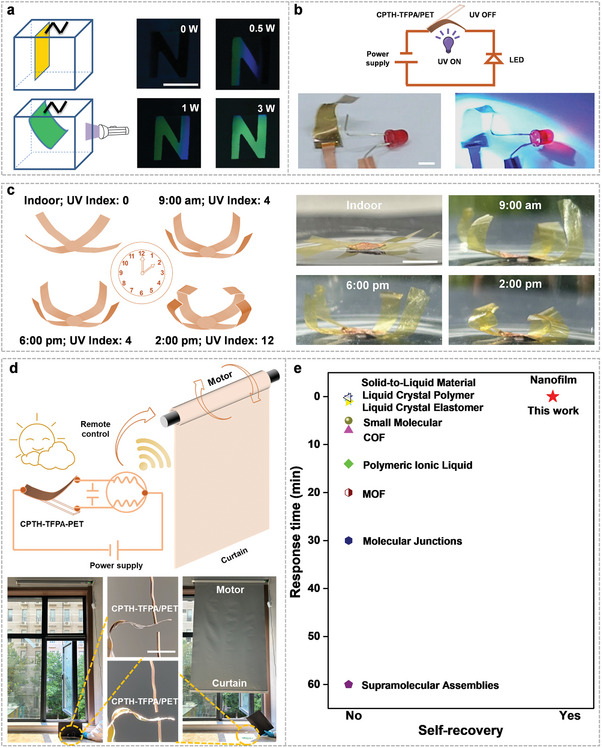
The CPTH‐TFPA/PET composite membrane acts as a visual sensor of UV light and a smart switch in practical scenarios. a) Schemes of the systems and the corresponding photos showing on‐demand display of the fluorescent letter “N” by varying the illuminating UV‐light power. b) Circuit diagrams and corresponding photographs of the smart electricity switch to turn the light‐emitting diode on/off in response to UV light. c) The photos of an artificial flower indoors without UV light illumination and outdoors at 9 am, 2 pm, and 6 pm under sunlight. The UV illuminances of sunlight is 0.02, 11.54, 31.42, and 9.33 mW cm^−2^. d) Circuit diagrams and corresponding photographs of the smart electricity switch to control the curtain open/close with/without the sunlight. The UV illuminance of sunlight is 30.35 mW cm^−2^. e) The UV response performance of the nanofilm in comparison with other materials.^[^ ^29^, ^30^, ^35^, ^36^, ^37^, ^52^, ^53^, ^55^, ^57^, ^58^ ^]^ The scale bars in all the digital photos are 1 cm.

The composite membranes can also be used for visualized sensing of UV light. To demonstrate this application, a fluorescent display system was constructed (**Figure** **4**a). One end of the fluorescent CPTH‐TFPA/PET membrane was suspended from the top panel on the left side of the “N” shaped display window. A black paper was placed at the bottom as a background. In the absence of UV light, the fluorescent membrane remained upright, displaying a black background. When exposed to 0.5 W of UV light, the composite membrane bent to cover part of the hollow area, revealing a green fluorescent number “1”. Similarly, patterns of “Λ” and “N” appeared when 1 and 3 W of UV light were used to irradiate the composite membrane (Figure 4a; Video S4, Supporting Information). Upon removal of the UV light, the patterns returned to their initial state. The CPTH‐TFPA/PI composite membrane also demonstrated photo‐controllable shape transformation from “J” to “U” and then to “Δ” (Figure S21 and Video S5, Supporting Information). Additionally, the CPTH‐TFPA/PET composite membrane enabled caterpillar‐like motions (Figure S22, Supporting Information). An asymmetric caterpillar‐like structure was prepared using the composite membrane, with slightly different angles at the two ends to ensure different contact areas on the surface. Upon exposure to UV light, the “caterpillar” bent into a semicircle shape but relaxed to an arch shape after switching off the UV light, inducing forward motion due to uneven friction of the two ends (Video S6, Supporting Information). The step‐size of the crawling motion could be controlled by adjusting the intensity of the UV light, paving the way for applications in photo‐driven soft robots. Furthermore, a smart switch was created by coating a thin gold film on both sides of the composite membrane. When illuminated by UV light, the membrane bent downward, resulting in the activation of a light‐emitting diode (LED) due to the completion of the electric circuit. Removal of UV light turned off the LED (Figure 4b; Video S7, Supporting Information).

### 2.5 Artificial Flower and Smart Switch Controlled by Sunlight

The further conceptual applications of the composite membrane were examined under sunlight that is the main source of UV irradiation, which include: 1) the fabrication of a highly sensitive and super‐fast UV‐responsive artificial flower was designed to monitor UV light intensity at different times of the day (Video S8, Supporting Information). As depicted in Figure 4c, the flower maintained a nearly flat posture indoors, but displayed a diverse range of postures when exposed to sunlight at 9 am, 2 pm, and 6 pm on a summer day; 2) another smart UV light‐responsive switch was developed, enabling the automatic opening and closing of a curtain by controlling the motor's forward and reverse rotation (Figure 4d; Videos S9 and S10, Supporting Information). These results illustrate the potential of the UV light‐responsive nanofilms for practical applications due to their unique super‐fast response speed and self‐recovering ability as compared to other UV‐responsive materials (Figure 4e).

### 2.6 Plausible Mechanism for UV Response

**Figure 5:**
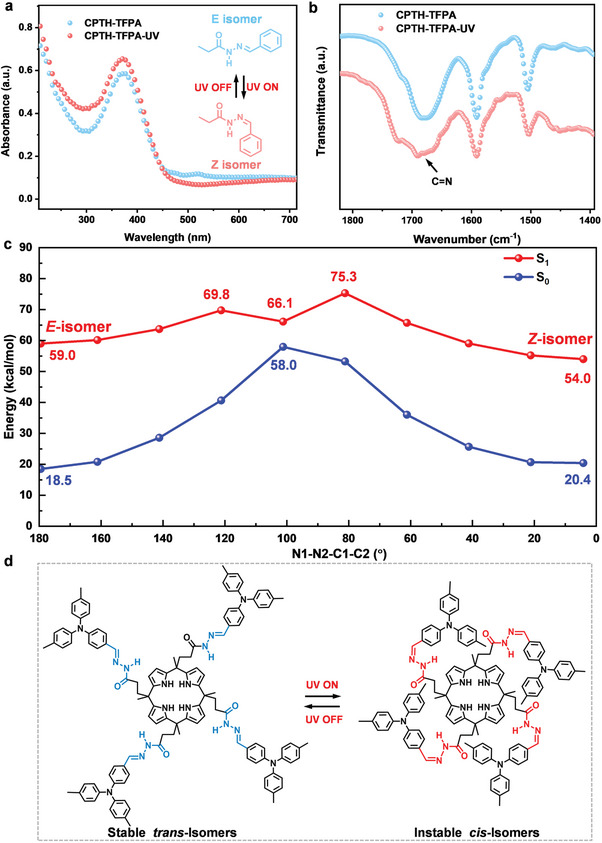
Investigation of the photo‐deformable mechanism of the CPTH‐TFPA nanofilm. a) Solid‐state UV–vis absorption spectra of the nanofilm before and after 2 hours high‐power UV light (300 W Xe lamp) irradiation, where the inset shows possible isomerization of the acylhydrazone bond. b) FTIR spectra of the CPTH‐TFPA nanofilm with and without UV irradiation. c) TD‐B3LYP/6‐31G^*^ calculated energy profiles of the CPTH‐TFPA model unit along with the N1‐N2‐C1‐C2 dihedral angles at the S_1_ state. d) Proposed isomerization of the CPTH‐TFPA nanofilm under UV light irradiation (375 nm).

Solid‐state absorption techniques were utilized to investigate the UV response mechanism of the nanofilms. The results in **Figure** **5**a show a significant increase in strong absorption ≈375 nm in the CPTH‐TFPA nanofilm and a notable decrease in weak absorption ≈520 nm after 2 h of irradiation with a powerful UV light at 375 nm, indicating that the acylhydrazone bonds undergo trans‐cis isomerization.^[^ ^52^ ^]^ The other two nanofilms exhibited similar properties (Figure S23, Supporting Information). Additionally, the characteristic FTIR signal at 1679 cm^−1^, associated with the bending vibration of the C═N group, shows a significant shift and change, further confirming the photo‐induced trans‐cis isomerization (Figure 5b).

To gain a deeper understanding of the isomerization of the acylhydrazone bonds in the nanofilms, theoretical calculations at B3LYP/6‐31G^*^ and TD‐B3LYP/6‐31G^*^ levels were performed. A structural model for the CPTH‐TFPA nanofilm was constructed first, which consists of one CPTH unit and one TFPA moiety (Section S7, Supporting Information). It is known that there are two configurations for the model, that is*, E*‐isomer and *Z*‐isomer. The *E*‐isomer of the CPTH‐TFPA model contains an in‐plane C═N bond (N1‐N2‐C1‐C2: 179.9°, Figure S24, Supporting Information), while the *Z*‐isomer adopts a twisted structure (N1‐N2‐C1‐C2: 5.8°, Figure S25, Supporting Information). For the *E*‐isomer, the vertical excitation energy to the S_1_ state is calculated to be 3.25 eV (381 nm, Table S1) at the Franck‐Condon point, which is close to the experimental value (375 nm, 3.31 eV). The maximum absorption peak of the *E*‐isomer is predicted to be 3.28 eV (378 nm) at the S_2_ state, which is nearly degenerate with the S_1_ state. The electronic states of them are different, where the S_1_ can be referred as a charge transfer state from the CPTH unit to the TFPA part (Figure S26, Supporting Information, HOMO→LUMO, 97.8%). For the S_2_ state, however, it mainly involves the local excitation within the TFPA unit, which explains why the oscillator strength is large (Table S2 and Figure S27, Supporting Information).

To explore the mechanism of the isomerization, both light and thermal driven pathways were considered (Figure 5c; Figure S28, Supporting Information). As seen, the isomerization is difficult to occur under thermal conditions due to the unfavorable kinetics (ca. 39.1 kcal mol^−1^). Upon excitation, the S_1_ state of the *E*‐isomer is populated, followed by the isomerization reaction. As shown in Figure 5c, the rotation of the N1‐N2‐C1‐C2 dihedral angle needs to overcome an energy barrier of 10.8 kcal mol^−1^ at 121.2°, and then reaches the intermediate state (101.2°). It should be noted that this intermediate state is close to the conical intersections of S_1_ and S_0_ states, which means it can hop to the ground state via the nonradiative transition. This is consistent with the experimental observations that the deformed nanofilm will return to its initial state without the need of another stimulus. In other words, only partial isomerization occurs during the UV‐induced morphological change of the nanofilms.

Clearly, the morphological change of the nanofilms upon UV irradiation must be a result of dramatically changed structures of the connected building blocks induced by the isomerization of the linking bonds (Figures 1b and 5d).^[^ ^40^, ^43^, ^53^ ^]^ Unlike most reported UV‐induced trans‐cis isomerization molecular systems,^[^ ^53^, ^54^ ^]^ the photo‐induced structural change of the nanofilms produced is completely reversible. This can be attributed to two main reasons. First, the trans‐isomer of the acylhydrazone bonds is thermodynamically more stable, which means that the UV‐induced *cis‐*structures tend to return to the initial trans‐state once the UV light is switched off. Second, the release of inherent tension in the nanofilm networks, accumulated during the photo‐induced trans‐cis isomerization process, promotes favorable structural changes after the UV light is switched off. Comparatively, other studies have also observed automatic *cis–trans* changes in molecular systems, but with much longer recovery times (up to 290 min) and only in a solution state.^[^ ^40^ ^]^ However, careful examination of the documented irreversible UV‐induced trans‐cis isomerization systems reveals that these systems generally possess hydroxy or methoxy moieties near the acylhydrazone bonds of interest, allowing for the formation of six‐membered rings.^[^ ^43^ ^]^ It is this intramolecular ring formation that not only promotes the trans‐cis isomerization but also stabilizes the *cis‐*isomers. Consequently, it is the “instability” of the *cis‐*isomers and the constrained state of the acylhydrazone bonds that may endow the nanofilms with the unprecedented reversible UV‐responsiveness in the solid state (Figure 5d).

The shape transformation or macroscopic motion is ascribable to the asymmetrical internal stress distribution in the CPTH‐TFPA/PET composite membrane. This is because, unlike the nanofilm, the supporting membrane, PET or PI, is not responsive to UV light, but stores the chemical energy converted from the UV light via observable shape change. Once the UV light was switched off, the stored energy was immediately released, driving the membrane back to its initial state, laying the foundation for UV detection, and addressing the unrecoverable shortcoming of most photo‐deformable materials.^[^ ^55^, ^56^, ^57^, ^58^ ^]^

## 3 Conclusion

In summary, we have successfully created a high‐performance UV detector based on the fast, robust, and fully reversible UV‐responsive nanofilms. The response and recovery times are both < 0.3 s, with the response range of 2.85 µW cm^−2^ to 8.30 mW cm^−2^. Additionally, our UV detector has shown exceptional stability, as > 500 consecutive tests yielded no observable changes in performance. Importantly, the detection wavelength of the UV light can be largely adjusted by modifying the structure of the photo‐responsive building block of the nanofilms. Furthermore, we have successfully achieved visual detection of UV light, enabling on‐site, real time and power free sensing. Moreover, the composite membrane can serve as sunlight‐responsive smart actuators for practical applications. The innovative CPTH‐TFPA/PET membrane and our sensing model provide new opportunities for flexible organic materials to achieve fast, sensitive, and stable UV detection.

## 4 Experimental Section

### Synthesis of CPTH

In a round‐bottomed flask, a specific quantity of methyl levulinate (1.5 equiv) and pyrrole (1 equiv) was dissolved in dry CH_2_Cl_2_ under N_2_. Next, 3 equiv of tetra‐methylammonium chloride was added, followed by the slow addition of 3 equiv of HCl (4.0 m in dioxane) to the flask over a period of 20 min. The reaction mixture was then stirred for 30 min while immersed in an ice‐water bath. Afterward, the flask was stirred in the dark under N_2_ for another 72 h at room temperature. The resulting mixture was filtered and the filtrate was diluted with CH_2_Cl_2_, then it was washed three times with saturated NaCl solution. The product was finally dried with Na_2_SO_4_, filtered, and concentrated in a vacuum. The black solid obtained was further purified by column chromatography (silica gel, *n*‐hexane/ethylacetate: 4/1), yielding a white solid that was fully dried at 50 °C in a vacuum oven. Subsequently, the white solid (2 g, 2.3 mmol) was dissolved in 60 mL methanol in a flask and excess hydrazine hydrate (80%, 5 mL, 80 mmol) was added to the solution. The system was heated to 70 °C and stirred for 3 days. After cooling to 25 °C, the mixture was concentrated under vacuum. Deionized water (pH 7.0) was added to the resulting solution, and sonicated for 30 min. The mixture was then centrifuged at 9000 r min^−1^ for 15 min to obtain a white solid, which was washed with deionized water three times to remove any remaining hydrazine hydrate. The final product was obtained after drying the resulting solid at 65 °C under vacuum (yield: 1.3 g, 65%).

### Synthetic of the Nanofilms and Composite Membrane

The nanofilms were prepared using a confined dynamic condensation method at the air/DMSO interface. Initially, CPTH and TFPA were mixed in DMSO at a 3:4 molar ratio. The resulting solution was then placed onto a pre‐washed 2 cm × 2 cm glass plate. The glass plate was treated with a plasma cleaner (air) for 3 min to ensure even distribution of the solution on the substrate surface. Subsequently, the sample was incubated for 2 h at room temperature in a constant temperature and humidity (≈50%) incubator. The nanofilms obtained were floated on Milli‐Q water and rinsed with water to remove any nanoparticles at the bottom. The freshly prepared CPTH‐TFPA nanofilm was then laminated onto commercially available PET or PI membrane and air‐dried at room temperature. Finally, the composite membrane was cut into 2 cm × 0.5 cm strips for UV detection. The interfacial adhesion strength between the nanofilm and the PET membrane was examined using an AFM‐based wear resistance test (Section S5, Supporting Information).

### Fabrication of UV Detector

Figure 1d and Figure S4 (Supporting Information) display both the schematic diagram and photographs of the UV detection system. It can be observed that one end of the composite membrane was securely attached to the bottom of the cuvette, while the other end remained unfixed. The laser displacement sensor was positioned at a distance of 30 cm from the membrane. The LED photodiodes, composite membrane, and laser beam were all positioned at the same height, ≈0.5 cm from the top of the suspended composite membrane. The software accompanying the laser displacement sensor was utilized to record the movement of the membrane. The power of the UV light source was controlled through an external resistance, while the UV power density was calibrated using an optical power meter.

### Statistical Analysis

The results presented in Figure 3d were obtained from three parallel tests with different CPTH‐TFPA/PET membranes, the corresponding SD is < 0.051. GAUSSIAN16 software was used for theoretical calculation.

## Conflict of Interest

The authors declare no conflict of interest.

## Supporting information

Supporting Information

Supplemental Video 1

Supplemental Video 2

Supplemental Video 3

Supplemental Video 4

Supplemental Video 5

Supplemental Video 6

Supplemental Video 7

Supplemental Video 8

Supplemental Video 9

Supplemental Video 10

## Data Availability

The data that support the findings of this study are available in the supplementary material of this article.
