# Supplementary material for: Fully Reversible and Super‐Fast Photo‐Induced Morphological Transformation of Nanofilms for High‐Performance UV Detection and Light‐Driven Actuators
Source: Adv Sci (Weinh). 2024 Jan 15;11(12):2307165. doi: 10.1002/advs.202307165 (PMC10966555; doi:10.1002/advs.202307165)
Supplement: Supplementary file 1 — Supporting Information [file ADVS-11-2307165-s009.pdf]

ADVANCED SCIENCE
Open Access

Supporting Information

for *Adv. Sci.*, DOI 10.1002/advs.202307165

Fully Reversible and Super-Fast Photo-Induced Morphological Transformation of Nanofilms for High-Performance UV Detection and Light-Driven Actuators

Xiangquan Liu, Jiahui Hu, Jinglun Yang, Lingya Peng, Jiaqi Tang, Xiaohui Wang, Rongrong Huang, Jianfei Liu, Kaiqiang Liu, Tingyi Wang, Xiaoyan Liu, Liping Ding* and Yu Fang**

Supporting Information

Fully Reversible and Super-fast Photo-induced Morphological Transformation of Nanofilms for High-performance UV Detection and Light-driven Actuators

Xiangquan Liu, Jiahui Hu, Jinglun Yang, Lingya Peng, Jiaqi Tang, Xiaohui Wang, Rongrong Huang, Jianfei Liu, Kaiqiang Liu, Tingyi Wang, and Xiaoyan Liu, Liping Ding*, Yu Fang**

Contents

1. Reagents and materials..2
2. Synthetic of CPTH..3
3. Synthetic of the nanofilms and composite membrane..3
4. Nanofilm characterization..4
5. Interfacial adhesion strength between the nanofilm and PET membrane..5
6. Supplementary figures..6
7. Computational details..29

1. Reagents and Materials

Pyrrole (98%, J&K Technology), methyl levulinate (97%, 3A Chemical), hydrazine hydrate (98%, Guangdong Guanghua Sci-Tech Co., Ltd), 1,3,5-tri-(4-formylphenyl)- amine (TFPA, 98%, Zhengzhou Alfa Chemical Co., Ltd), 4, 4′ ,4″ ,4‴-(ethene-1,1,2,2- tetrayl)tetra-benzaldehyde (ETBA, 97%, Aladdin Chemistry Co., Ltd), 4, 4′ ,4″- ((1,3,5-triazine-2,4,6-triyl)tris(oxy))tribenzaldehyde (TOFB, 98%, Zhengzhou Alfa Chemical Co., Ltd), track-etched poly(ethylene terephthalate) (PET) nuclear pore membranes (thickness: 10 μm, Wuwei Kejin Xinfa technology Co. Ltd). Polyimide (PI) membrane (thickness: 3 μm, E. I. Du Pont Company). Glass plate (thickness: 1 mm, Corning Company). TEM grid (T10044, Beijing XXBR technology Co. Ltd). Milli-Q water (Milli-Q EQ 7000, Merck Company). LED UV source (375 nm, 30 W; 310 nm, 5 W; 254 nm, 5 W, Shenzhen Ruibao light technology Co. Ltd). Xenon lamp source (YDD-XE300, Xian Yado Photoelectric Technology Co. Ltd). Displacement sensor (sensitivity: 1 μm, measuring range: 10 mm, BOJKE technology Co. Ltd). Optical power meter (PM100D, Thorlabs).

2. Synthesis of CPTH

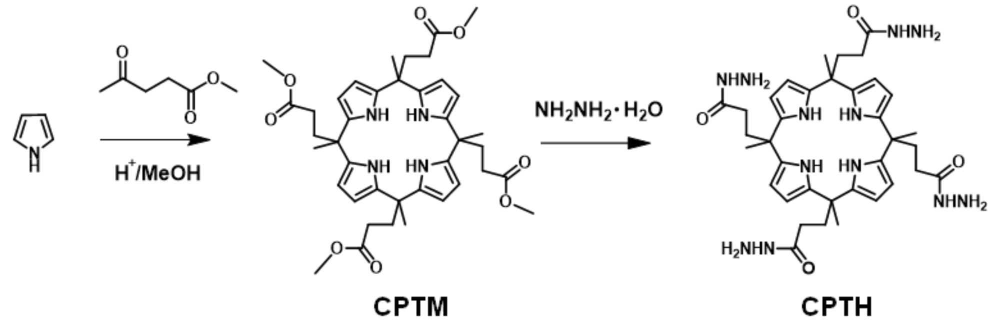

Scheme S1. The synthetic route of CPTH.

3. Synthetic of the Nanofilms and Composite Membrane

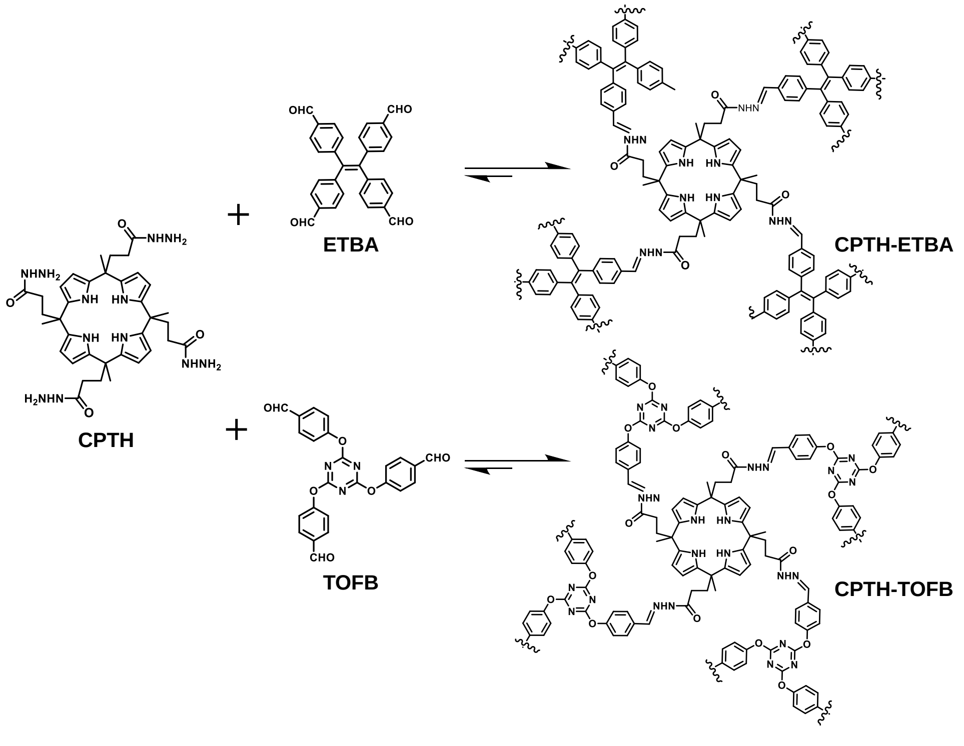

Scheme S2. Dynamic condensation reaction between the building blocks occurred in the formation of the CPTH-ETBA and CPTH-TOFB nanofilms.

4. Nanofilm characterization

Fourier transform infrared spectroscopy (FTIR, Bruker) and X-ray photoelectron spectroscopy (XPS, AXIS ULTRA from Kratos Analytical Ltd.) were utilized for the characterization of the chemical composition of the CPTH-TFPA nanofilms. The binding energies were calibrated by setting the C1s peak at 284.6 eV. The surface morphology of the nanofilms was analyzed using emission scanning electron microscopy (SEM, SU8020, Hitachi). The morphology and thickness of the nanofilms were determined by atomic force microscope (AFM, Dimension Icon Atomic force microscope (Bruker) in ScanAsyst mode). The Young's modulus (E) of the nanofilms was measured in fast force mapping mode by AFM (Cypher VRS, Oxford Instruments), employing an AFM cantilever (AC240TS-R3, Asylum Research Probes). The tip radius of the cantilever was obtained using a relative calibration method, with PSFILM-12M (E = 2.7 GPa, Bruker) utilized for the calibration. The estimated mean value of the tip radius was 7 nm. In all the samples investigated with Fast Force Mapping mode, a force of 50 nN was exerted during the measurements. The internal structure of the nanofilms was examined using a high-resolution transmission electron microscope (HETEM, Tecnai G2 F20) at 5 kV. The X-ray diffraction (XRD) patterns and contact angles of the nanofilms were measured using Bruker D8 Discover and a video-based contact angle measuring system (OCA20, Dataphysics), respectively. Lastly, a solid-state UV-vis absorption spectroscopy study was conducted using a UV-visible near-infrared spectrometer (Lambda 1050, PE Company).

5. Interfacial adhesion strength between CPTH-TFPA nanofilm and PET membrane

The interfacial adhesion strength between the nanofilm and PET membrane was investigated by wear resistance tests using Tap300DLC AFM cantilever in contact mode. The spring constant of the AFM cantilever is 36 N/m, and the tip radius is 14 nm. Contact pressure was obtained by dividing the applied force by the cantilever tip surface area ($2\pi r^2$). The applied forces were 400 and 800 nN, corresponding to contact pressures were 283 MPa and 566 MPa, respectively. The scan rate was 0.8 Hz, and the resulting wear region was then imaged by using AC mode. As shown in Figure S9, the worn area can be clearly noticed in the resulting images after the wear tests under the applied forces of 400 and 800 nN, and the worn material has accumulated at the edges of the worn areas. However, the indentation depth of the scanning region is in the range of 2 ~ 10 nm, that is much smaller that the thickness of nanofilm (~ 35 nm). This means that the nanofilm was not delaminated from the PET membrane under the high contact pressure of 566 MPa, indicating the interfacial adhesion strength between the nanofilm and the PET membrane is high. Moreover, there is no individual nanofilm layer peeled off under the lateral forces, indicating the adhesion force between the nanofilm is strong. This is likely attributed to the attraction forces including hydrogen bonds and van der Waals forces between the nanofilms.

6. Supplementary figures

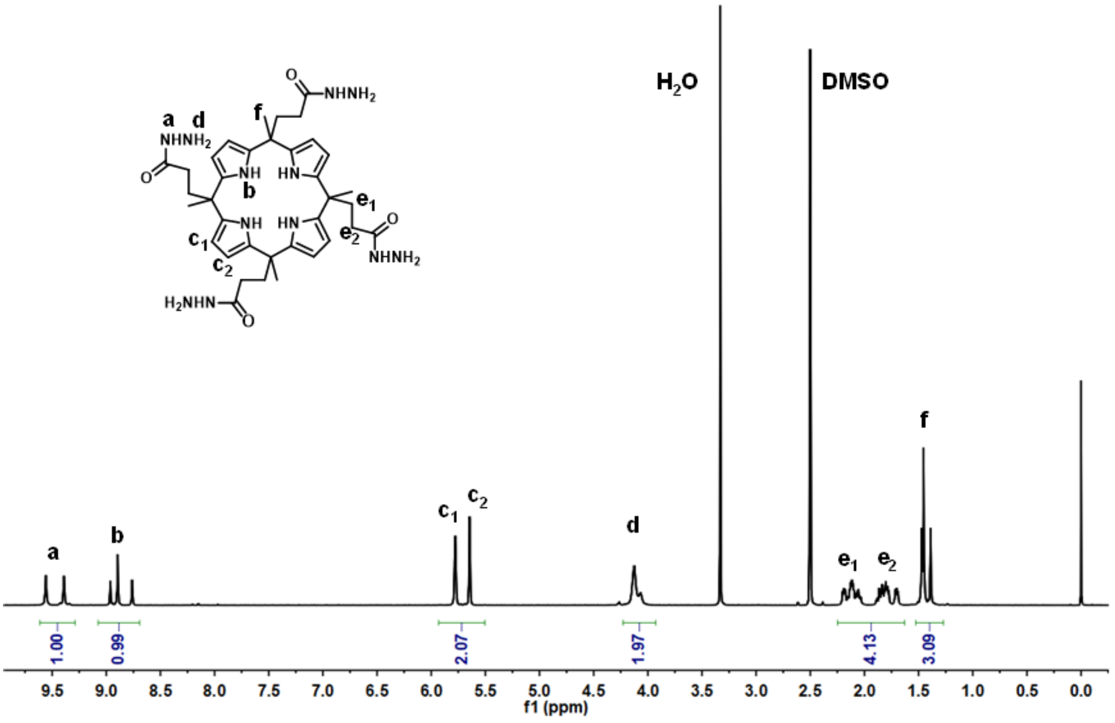

Figure S1. ^{1}H NMR spectrum of CPTH.

^{1}H NMR (600 MHz, DMSO) δ 9.43 (4H, m, -NH), 8.99 (4H, m, -NH), 5.93-5.50 (8H, m, pyrrole-CH-), 4.10 (8H, s, $-NH_2$), 2.25-1.63 (16H, m, $-CH_2CH_2-$), 1.53-1.27 (12H, m, $-CH_3$).

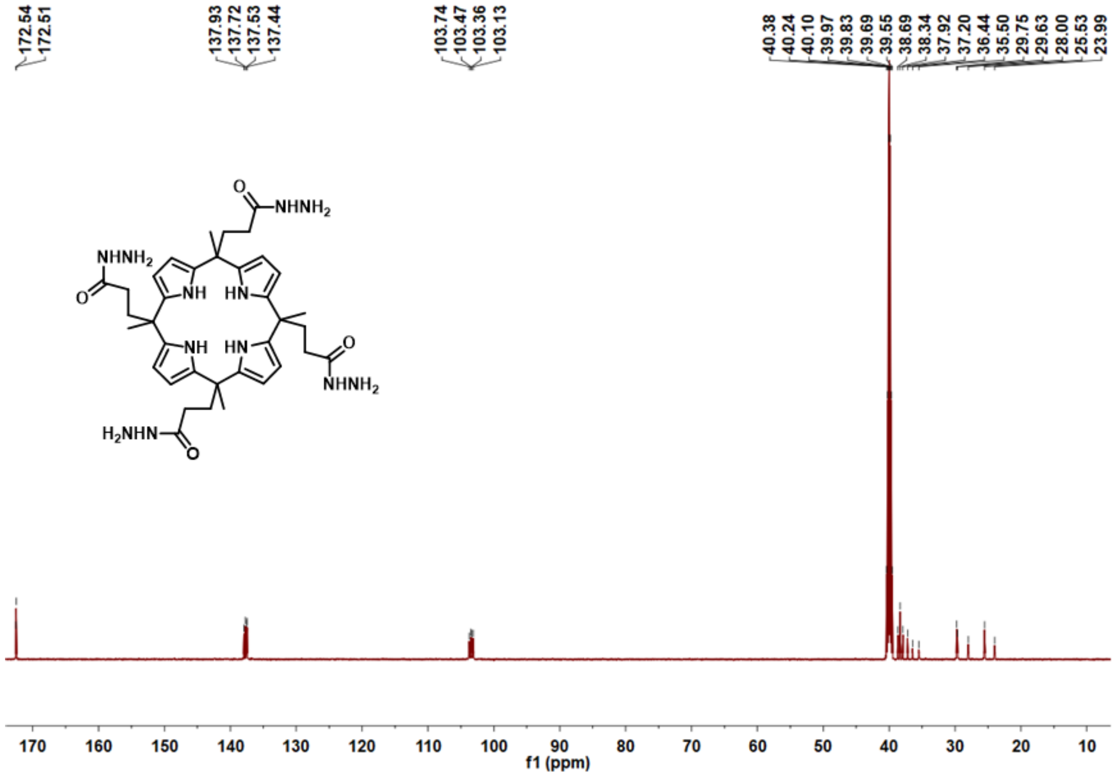

Figure S2. ^{13}C NMR spectrum of CPTH.

^{13}C NMR (151 MHz, DMSO) δ 172.54, 172.51, 137.93, 137.72, 137.53, 137.44, 103.74, 103.47, 103.36, 103.13, 40.38, 40.24, 40.10, 39.97, 39.83, 39.69, 39.55, 38.69, 38.34, 37.92, 37.20, 36.44, 35.50, 29.75, 29.63, 28.00, 25.53, 23.99.

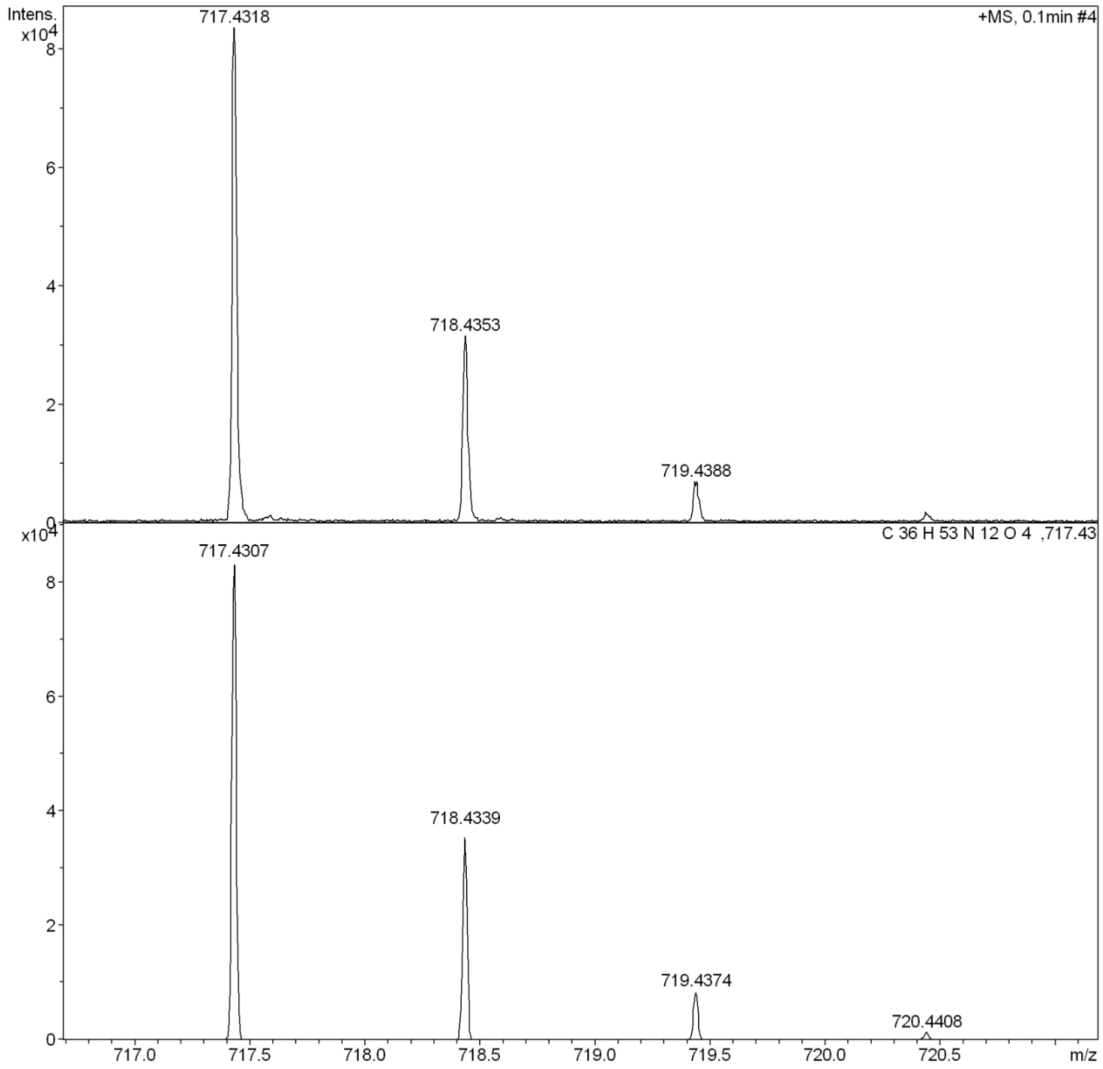

Figure S3. APCI mass spectrum of CPTH.

The HRMS (APCI) *m*/*z*: calcd for $C_{36}H_{53}N_{12}O_4$, $[M + H]^+$ = 739.4307, found 717.4318.

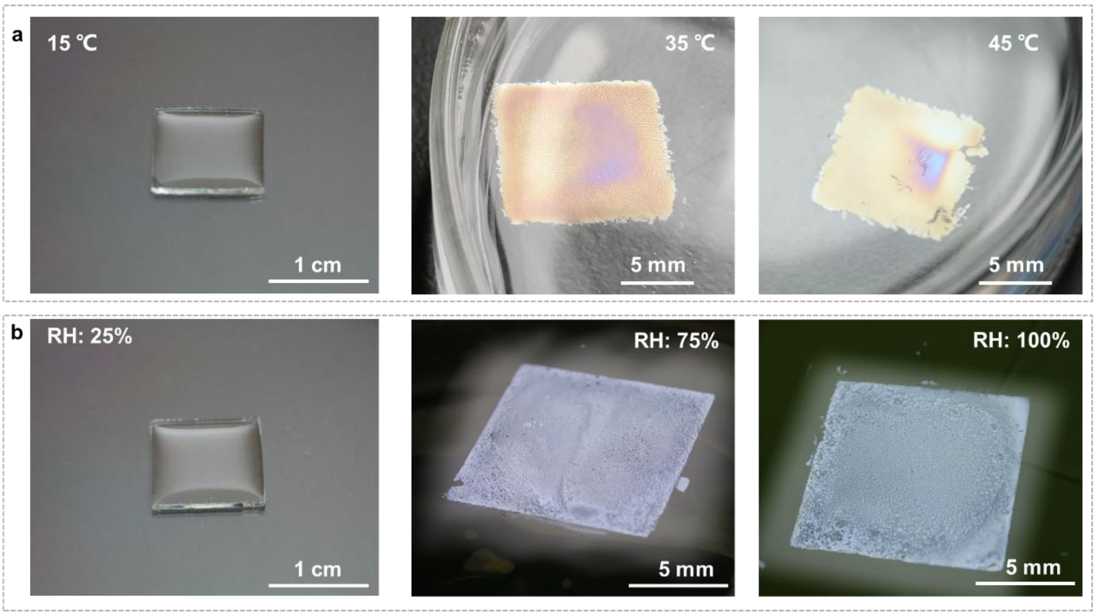

Figure S4. (a) Preparation of nanofilms under a relative humidity (RH) of 50% at 15℃, 35℃ and 45℃, respectively. (b) Preparation of nanofilms at 25℃ under the RH of 25%, 75% and 100%, respectively.

***Note*:** As shown in **Figure S4**, we found that at low temperature, the two monomers cannot react to form a membrane. At too high temperatures, the reaction between the monomers is too fast, resulting in an uneven film with poor robustness. The 50% humidity was employed because we observed that at lower humidity, monomers cannot accumulate at the air-DMSO interface, leading to the absence of a distinct nanofilm formation. This is due to there are fewer hydrogen bonds formed between water molecules and monomers. On the other hand, increasing humidity facilitates the accumulation of monomers, but it also introduces water molecules as catalysts in the reaction. This causes the monomer reaction rate to become excessively fast, resulting in uneven films and inferior mechanical properties.

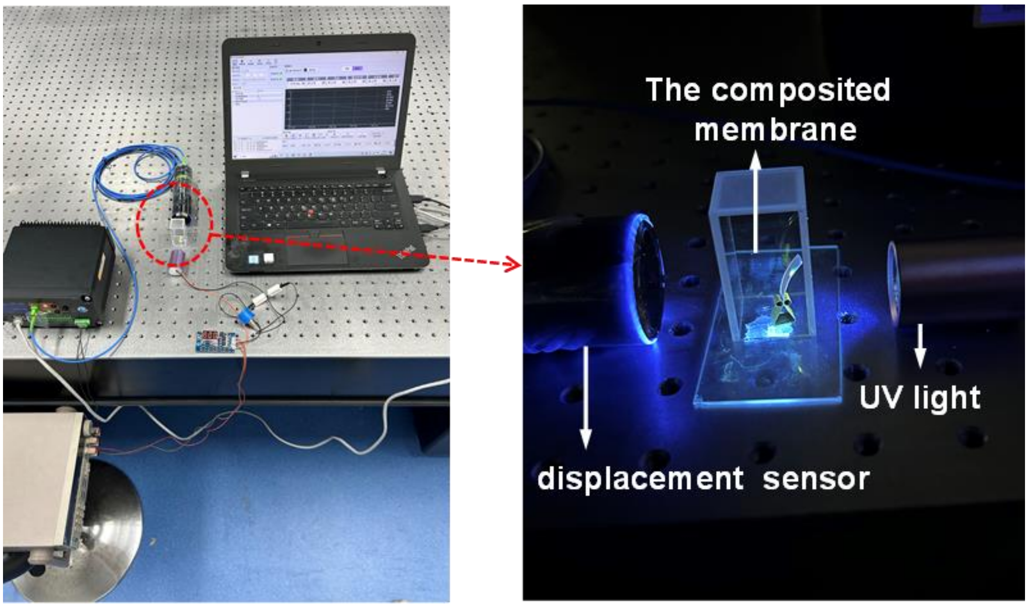

Figure S5. The photographs of the system for UV light detection and partially enlarged photographah while the system is working.

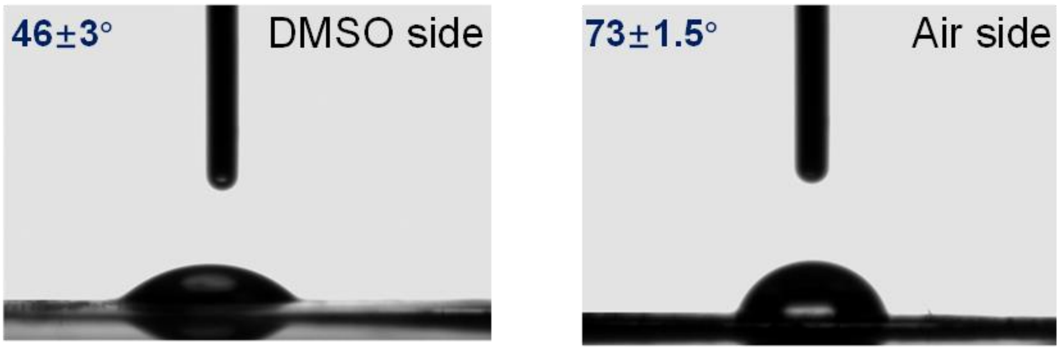

Figure S6. The contact angles of the two sides of CPTH-TFPA nanofilm.

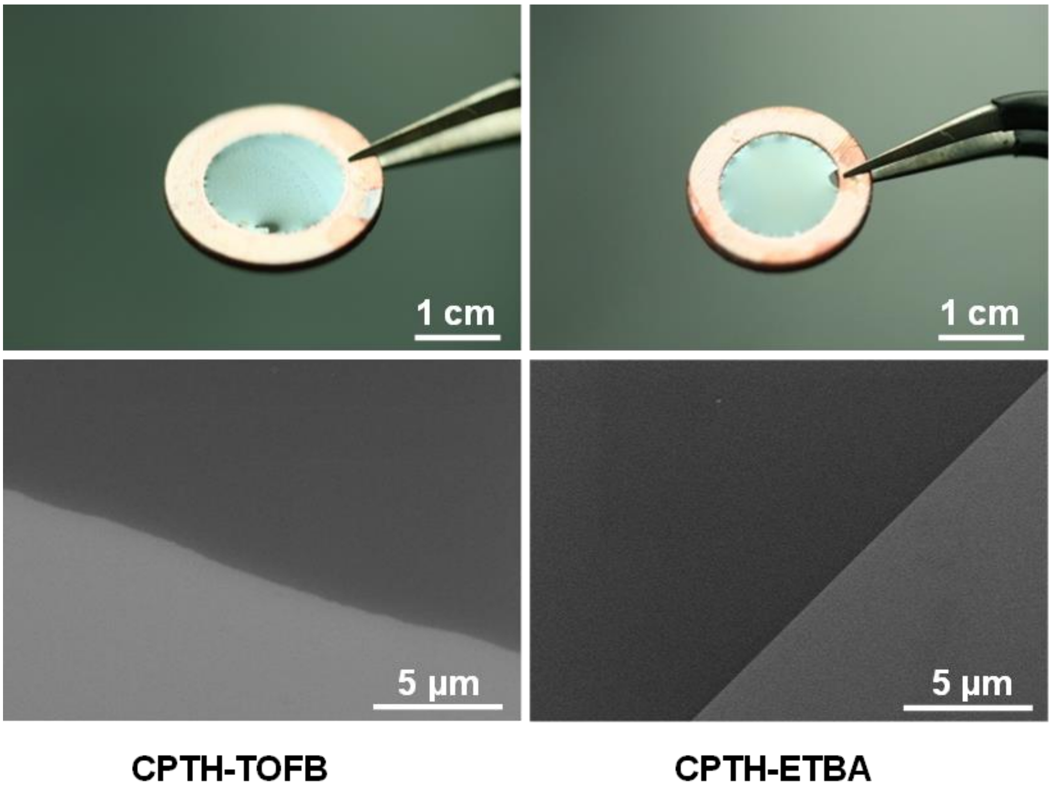

Figure S7. Photographs and SEM images of the CPTH-TOFB (left) and CPTH-ETBA (right) nanofilms formed at the humid air/DMSO interface (relative humidity ~ 50%).

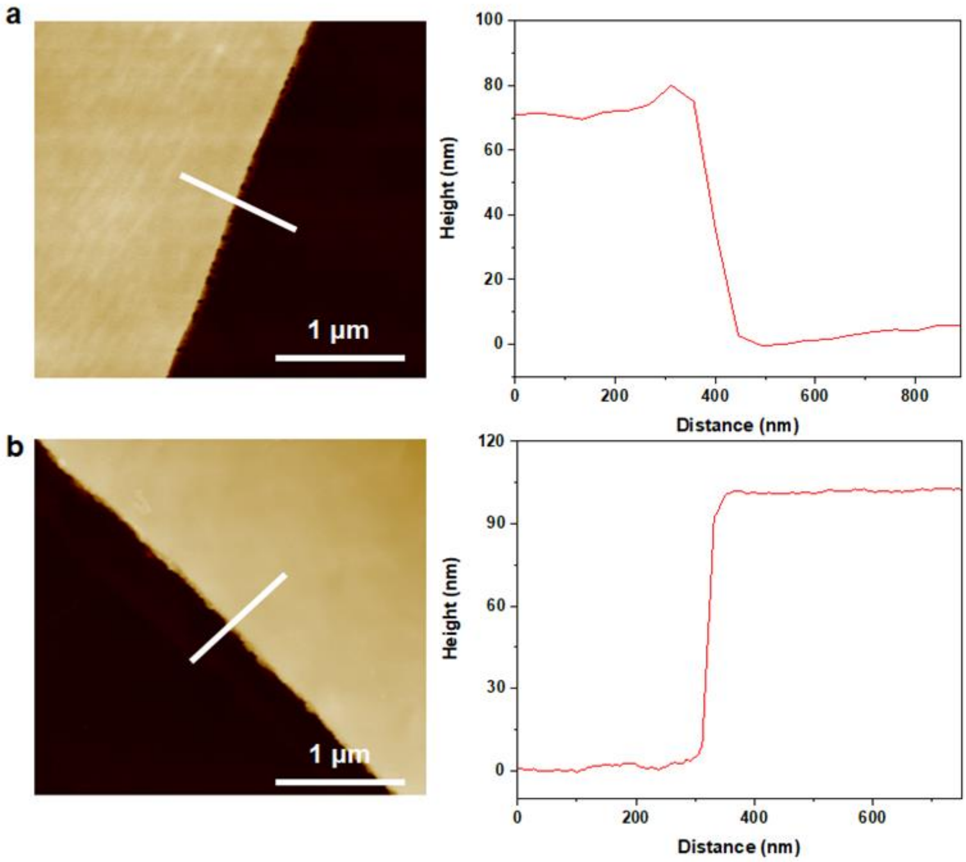

Figure S8. CPTH-TFPA nanofilms of different thicknesses prepared in the reaction systems of different precursor concentrations. *Note*: (a) 0.1 wt%, (b) 0.2 wt%.

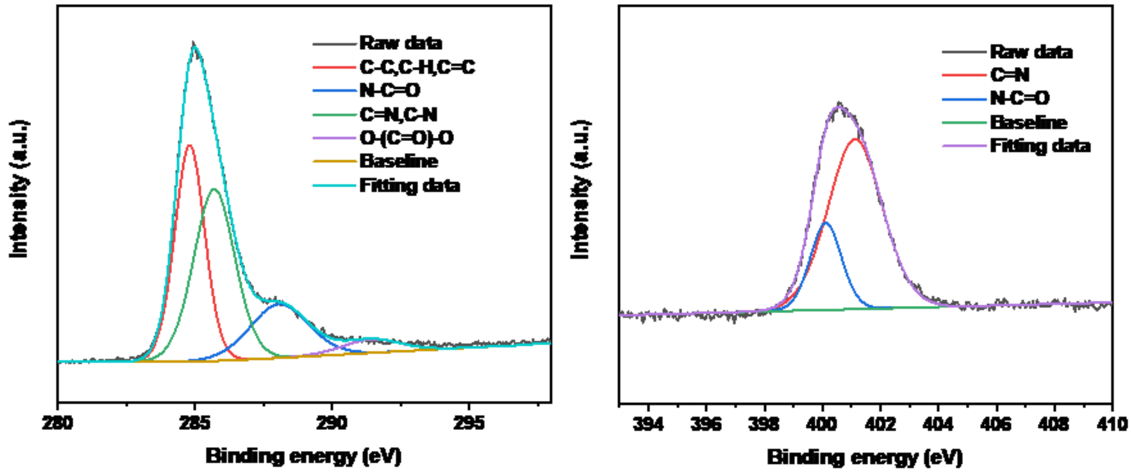

Figure S9. C 1s (left) and N 1s (right) XPS spectra of the prepared CPTH-TFPA nanofilm.

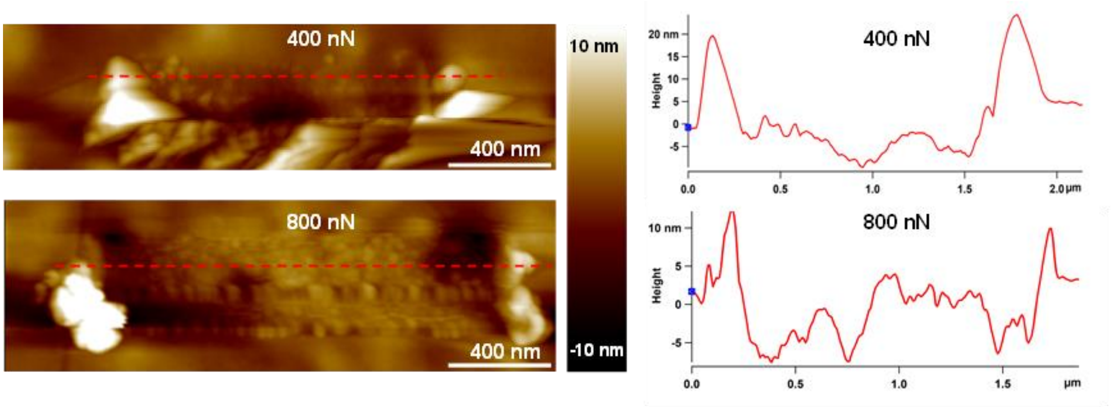

Figure S10. Wear experiments conducted with an AFM tip on the CPTH-TFPA/PET sample cured with 400 nN and 800 nN applied force.

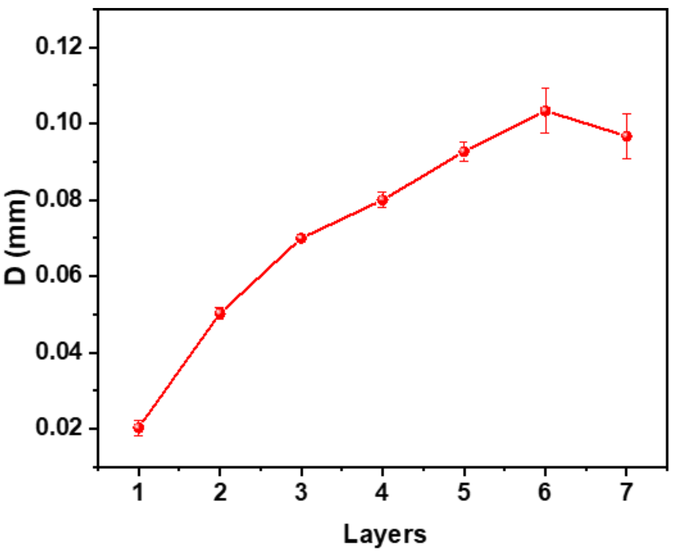

Figure S11. Thickness dependence of the UV-induced displacement of the fabricated CPTH-TFPA/PET composite membranes.

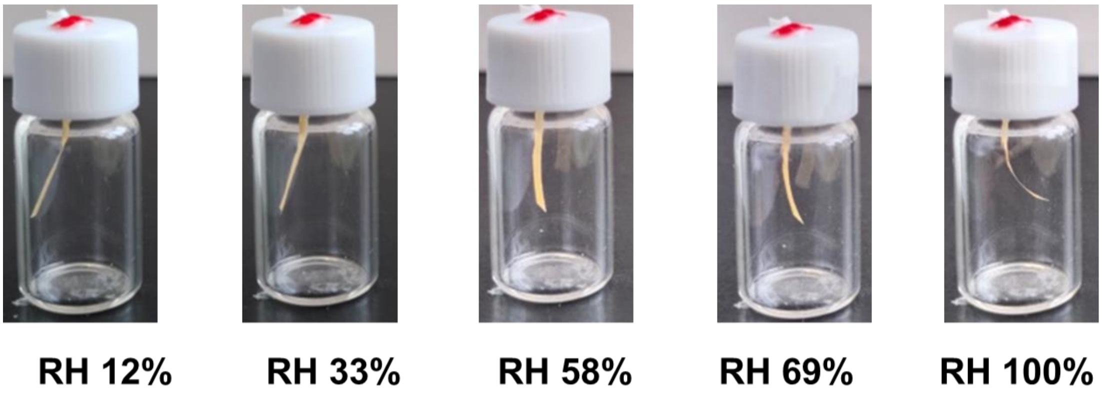

Figure S12. The CPTH-TFPA/PET composited membrane at different humidity.

Note: The experiment was realized by filling the vial with the vapor of a saturated solution of different salts (LiCl, $MgCl_2$, NaBr, CuCl) at 25 ℃.

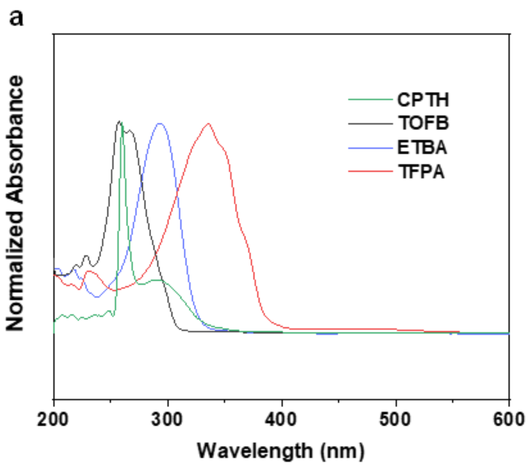

Figure S13. (a) Solid-state UV-vis absorption spectra of the building blocks and (b) the obtained CPTH-TFPA, CPTH-ETBA and CPTH-TOFB nanofilms.

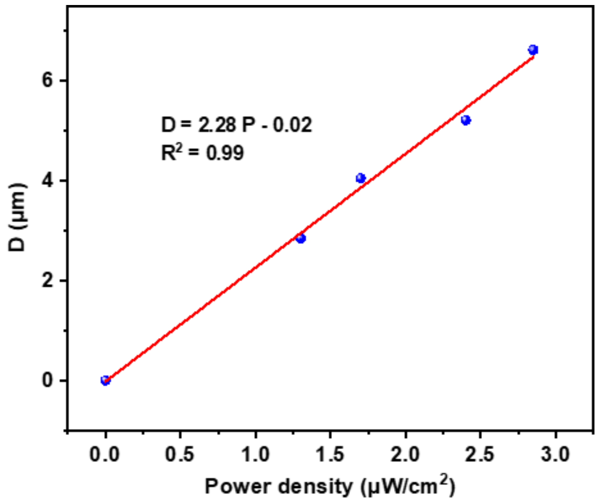

Figure S14. Experimental data and fitting curves of relative displacement changes of the prepared CPTH-TFPA/PET composite membrane versus UV power density at 375 nm.

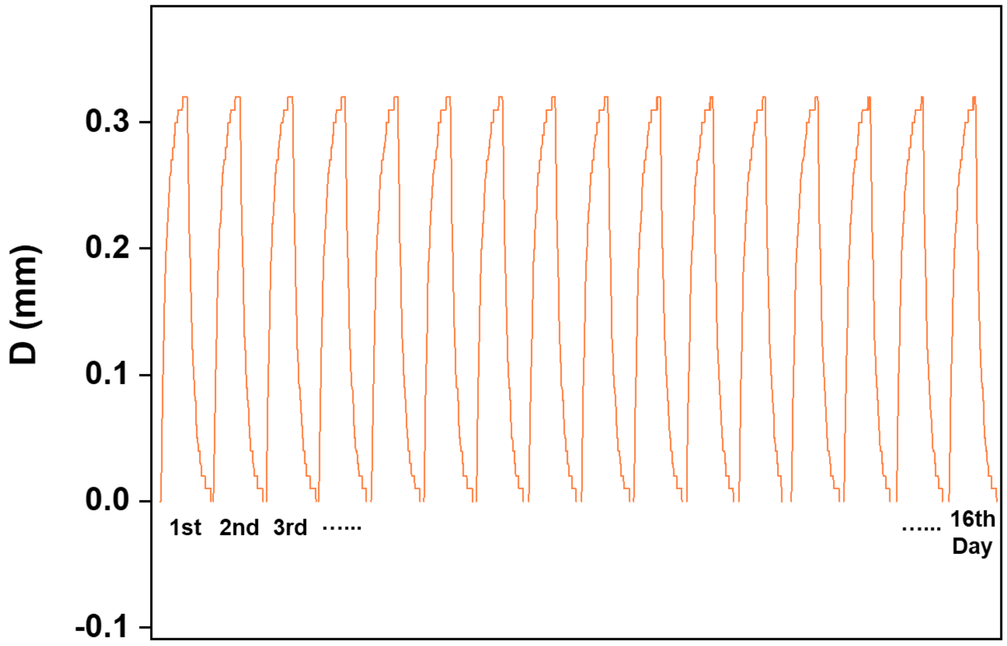

Figure S15. The responses of the membrane to UV light for 16 days (375 nm, 0.05 mW cm^{-2}).

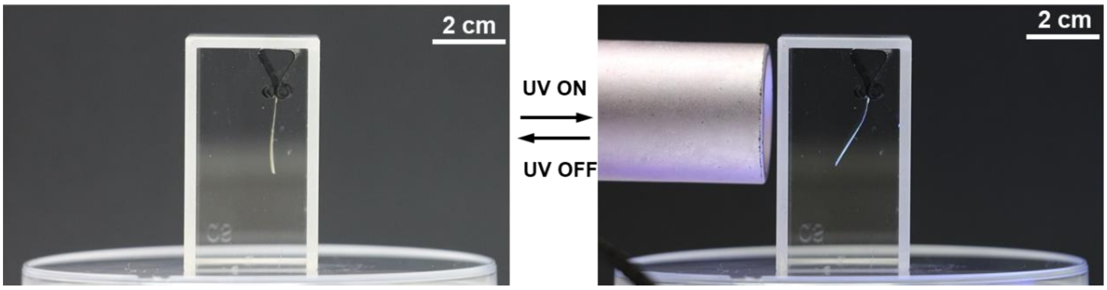

Figure S16. The UV light response of a CPTH-TFPA/PET membrane after a storage of about 6 months.

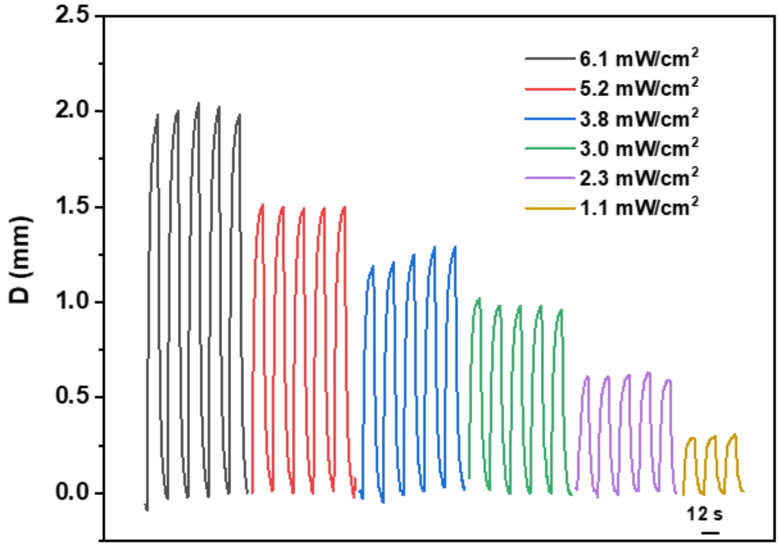

Figure S17. The response of CPTH-TOFB/PET composite membrane to 254 nm UV light at different illuminances.

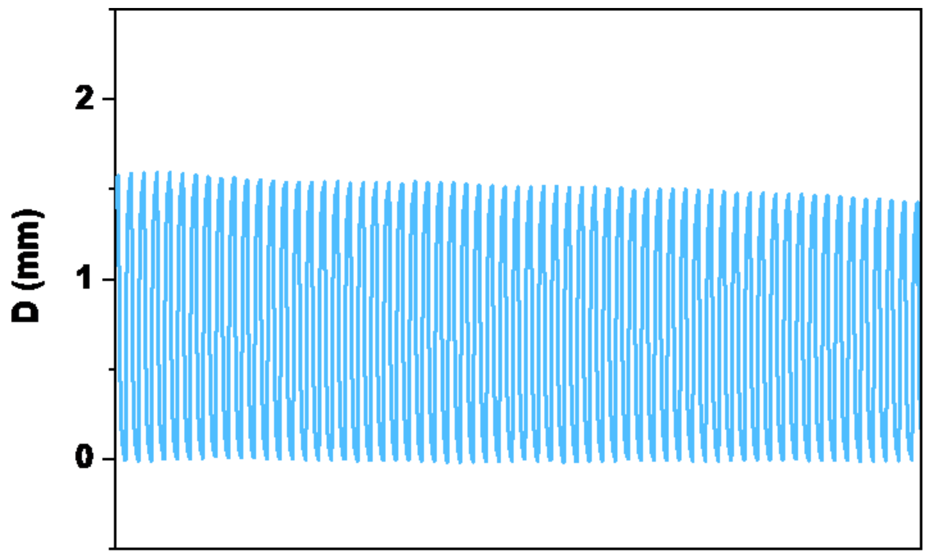

Figure S18. The response of the CPTH-TOFB/PET composite membrane to 254 nm UV light during consecutive 60 UV on/off cyclic operation upon 5.2 mW cm^{-2} UV light.

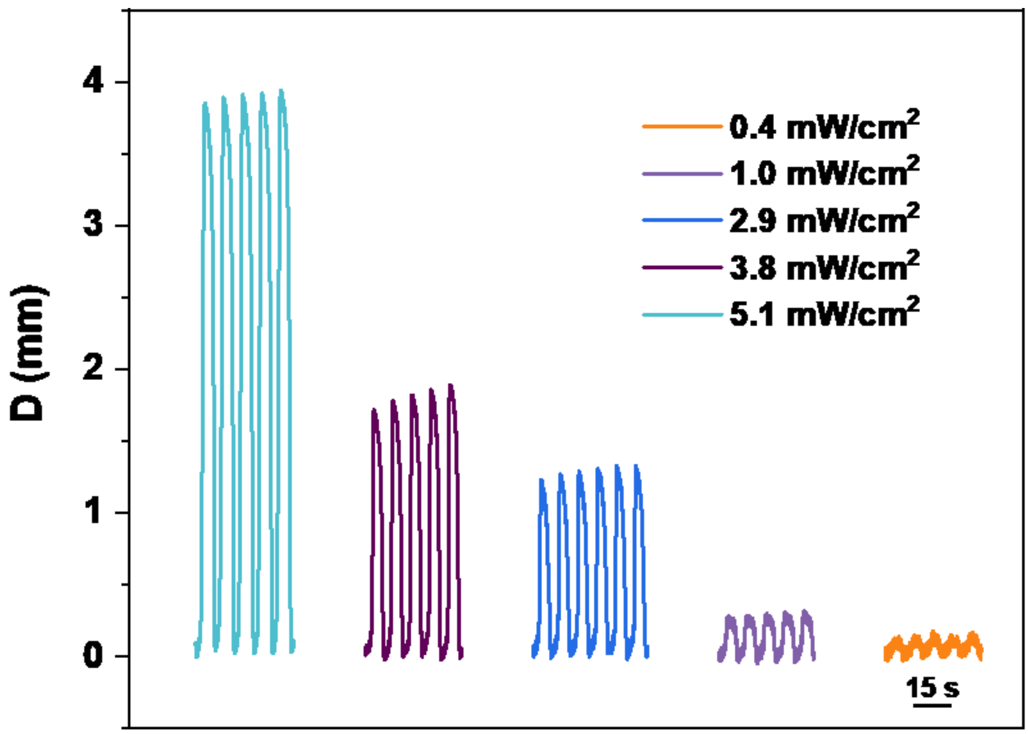

Figure S19. The response of the CPTH-ETBA/PET composite membrane to 310 nm UV light at different illuminances.

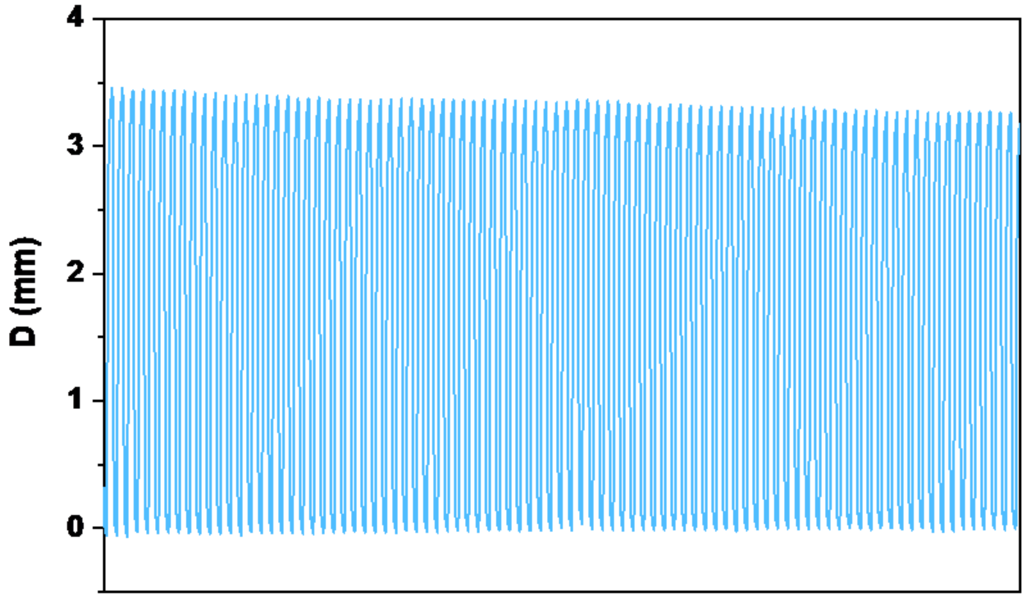

Figure S20. The response of the CPTH-ETBA/PET composite membrane during consecutive 80 UV on/off cyclic operation upon 4.5 mW cm^{-2} UV light.

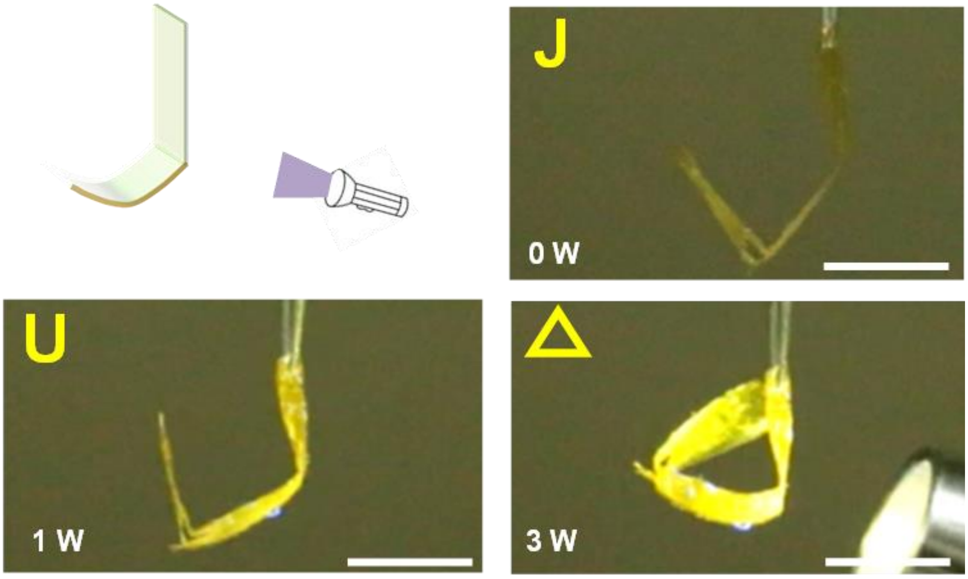

Figure S21. The photos of the designed letter ‘J’ formed by CPTH-TFPA/PI membrane under different UV-light power, where the scale bars in all the digital photos are 1 cm.

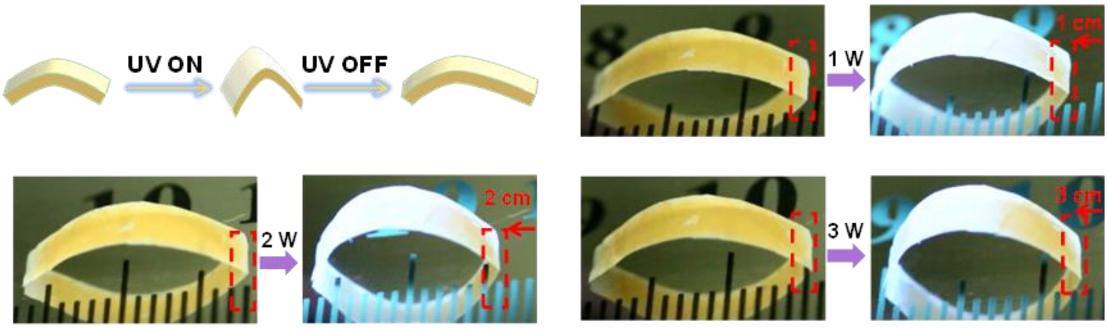

Figure S22. The photos of the designed letter ‘J’ formed by CPTH-TFPA/PI membrane under different UV-light power, where the scale bars in all the digital photos are 2 cm.

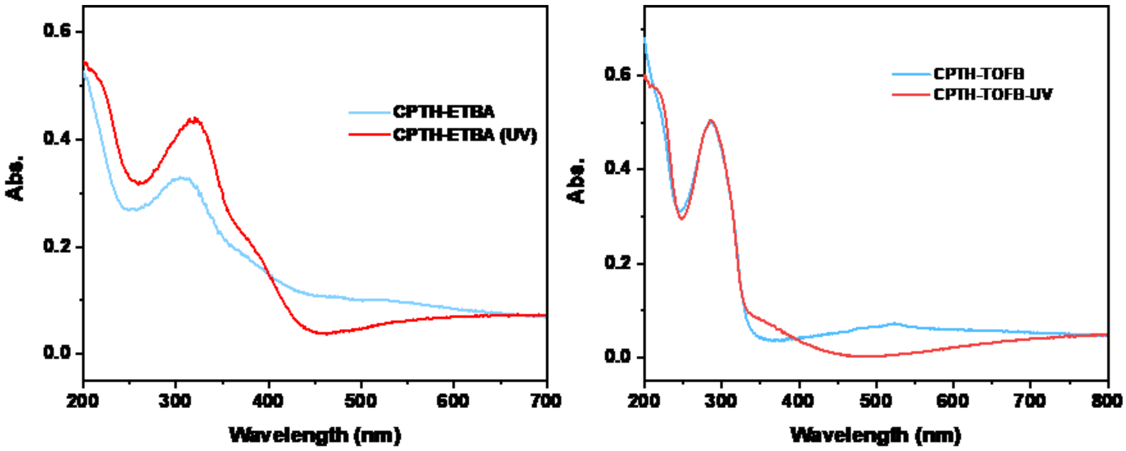

Figure S23. Solid-state UV-vis absorption spectra of CPTH-ETBA (left) and CPTH-TOFB nanofilms (right) before and after UV irradiation.

7. Computational details

Geometry optimizations are performed at the B3LYP and TD-B3LYP levels for the ground and excited states, respectively, using GAUSSIAN16 package.[1-5] The Grimme dispersion correction is carried out considering weak dispersion interactions.[6] In all the DFT and TD-DFT calculations, the 6-31G* basis sets are used.[7-8]

Table S1. Calculated vertical excitation energy ($E_{\perp}$, eV), wavelength (nm), main configurations, and oscillator strength of the *E*-isomer.

States	$E_{\perp}$ (eV)	Wavelength (nm)	Configurations	Weight	Oscillator strength
S_1	3.25	381	HOMO→LUMO	81.8%	0.2107
S_2	3.28	378	HOMO-1→LUMO	83.3%	0.7094
S_3	3.57	348	HOMO-2→LUMO	96.0%	0.0003
S_4	3.80	326	HOMO-1→LUMO+1	94.3%	0.0255
S_5	3.90	318	HOMO-2→LUMO	95.2%	0.0085

Table S2. Calculated vertical excitation energy ($E_{\perp}$, eV), wavelength (nm), main configurations, and oscillator strength of the *Z*-isomer.

States	$E_{\perp}$ (eV)	Wavelength (nm)	Configurations	Weight	Oscillator strength
S_1	3.23	384	HOMO→LUMO	95.1%	0.0034
S_2	3.37	368	HOMO-1→LUMO	97.5%	0.3591
S_3	3.65	340	HOMO-2→LUMO	94.5%	0.0008
S_4	3.83	324	HOMO-1→LUMO+1	93.9%	0.0131
S_5	3.91	317	HOMO-2→LUMO	89.4%	0.0022

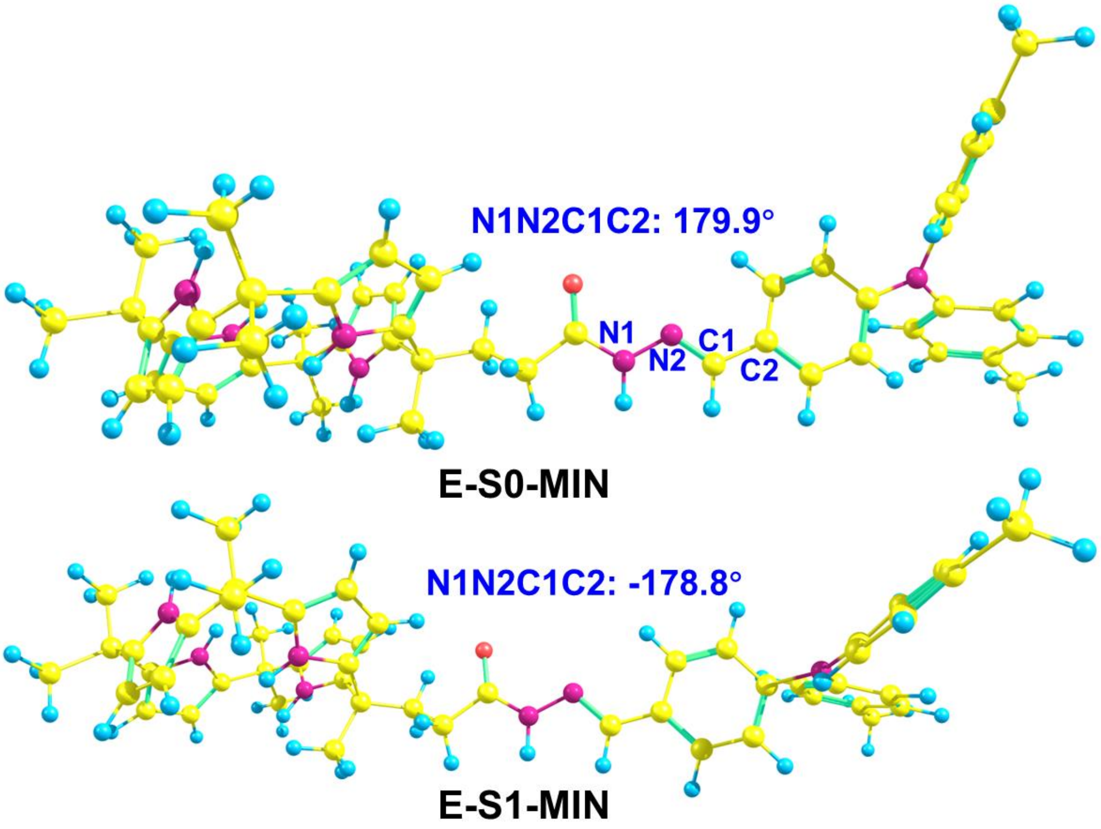

Figure S24. Optimized S_0 and S_1 structures of the *E*-isomer.

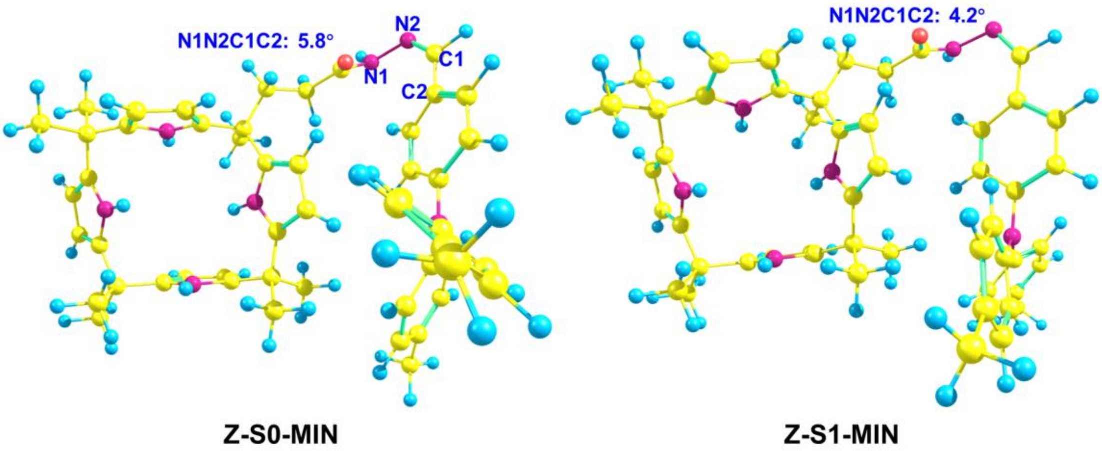

Figure S25. Optimized S_0 and S_1 structures of the *Z*-isomer.

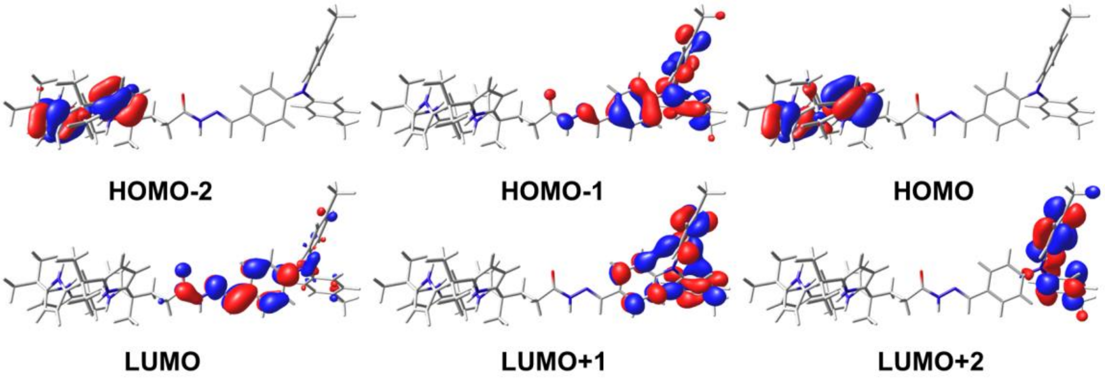

Figure S26. Frontier molecular orbitals of the *E*-isomer.

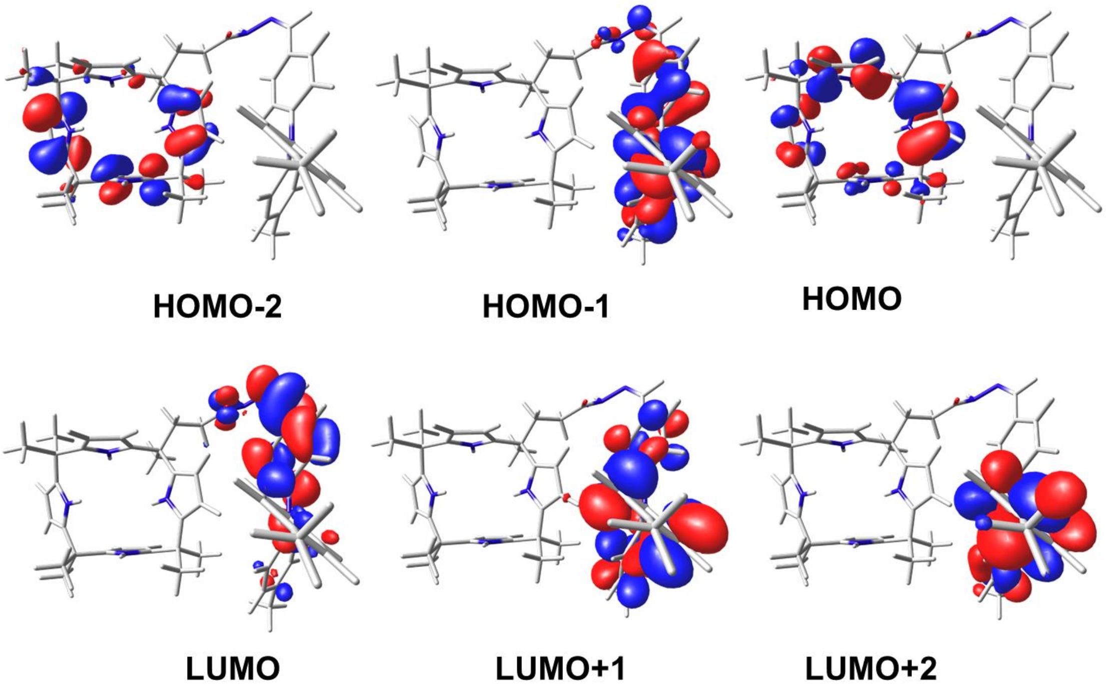

Figure S27. Frontier molecular orbitals of the *Z*-isomer.

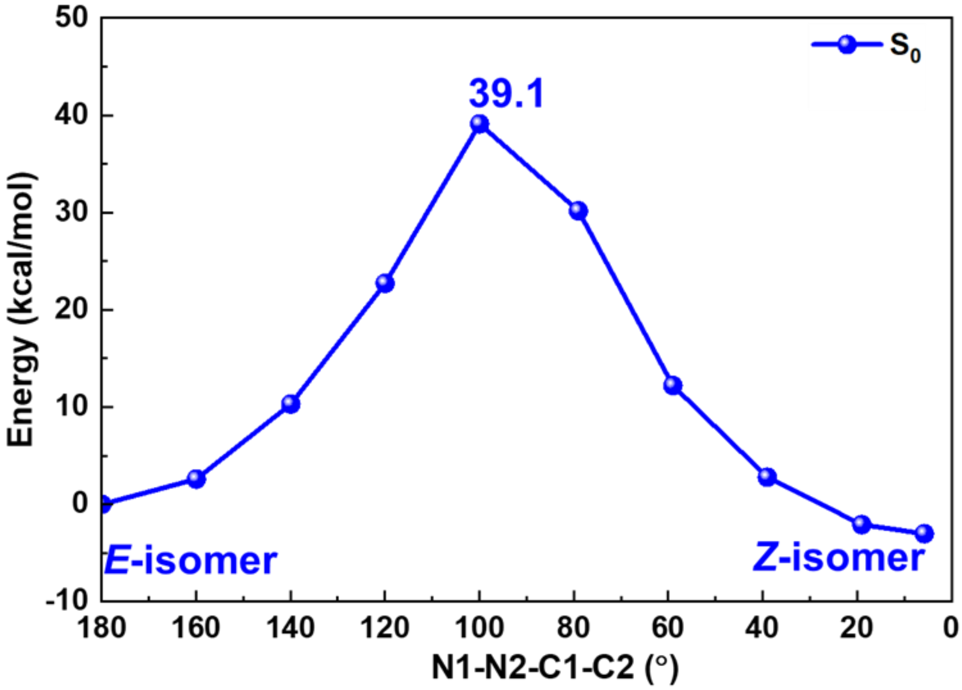

Figure S28. B3LYP/6-31G* calculated energy profiles of the CPTH-TFPA model unit along with the N1-N2-C1-C2 dihedral angles at the S_0 state.

Cartesian coordinates of optimized structures

***E*-isomer**

S_0

H	0.125205009	5.538913400	0.382012027
C	5.757486411	7.023916526	1.782981131
C	6.714523469	6.014484411	1.466717104
C	6.374749447	4.876634351	2.173601154
N	5.244255379	5.182683373	2.900034208
C	4.849694350	6.483591470	2.674406195
C	7.069434500	3.535465253	2.350424171
C	6.048589462	2.503242178	2.780795201
C	5.787775406	1.923926136	4.009756290
C	4.620758334	1.119057082	3.878046280
C	4.181498301	1.218049090	2.571772183
N	5.057330363	2.064870147	1.922946137
C	2.970171212	0.577002040	1.906427139
C	1.716469123	1.123001082	2.566517183
C	0.877890064	0.597181045	3.534424255
C	-0.070079005	1.605986116	3.879578280
C	0.206953015	2.726108198	3.118961223
N	1.287795090	2.410275171	2.324309168
C	-0.500343036	4.061282290	2.954792215
C	0.507976037	5.095727369	2.498578179
C	0.737423054	5.674067386	1.262842092
C	1.903257134	6.485244461	1.367157099
C	2.373066173	6.390971481	2.663085194
N	1.517897112	5.538586421	3.331504238
C	3.589620258	7.045185482	3.304865239
H	5.736610422	8.032629594	1.394347099
H	7.567704570	6.121215441	0.810350061
C	7.751914557	3.114482224	1.029357072
H	6.378044475	2.056799146	4.905704354
H	4.141404297	0.538652041	4.654233335
C	2.953202212	0.871140065	0.388569028

H	0.935124066	-0.404213029	3.941433285
H	-0.876964063	1.508030110	4.593253330
C	-1.608087113	3.919463284	1.883898137
H	2.358097172	7.070478514	0.579686043
C	3.525921255	8.568383616	3.059437223
H	4.974114357	2.361513170	0.963478071
H	1.623016119	5.247197377	4.290705308
H	4.757530343	4.502697323	3.467649252
H	1.738251127	3.086317220	1.723298123
C	3.059248222	-0.956375068	2.137243153
C	1.921697136	-1.778185129	1.502327108
C	1.595927112	-3.011366216	2.340899171
N	1.371976099	-4.153892298	1.594361113
N	1.004787070	-5.324732401	2.179420157
C	0.805680056	-6.329922471	1.403551100
O	1.510780106	-2.977307215	3.556290256
C	0.410123029	-7.637673550	1.913205138
C	0.192542014	-8.699533636	1.020631071
C	-0.193705014	-9.955553722	1.471812108
C	-0.362043026	-10.195500732	2.846906204
C	-0.135775010	-9.134018638	3.748355271
C	0.236003017	-7.881457571	3.289652239
N	-0.746068054	-11.467968812	3.316683238
C	-0.314017022	-12.646281918	2.646418190
C	-1.581051114	-11.593872833	4.462370323
C	1.018909076	-12.787464927	2.233556159
C	1.432911103	-13.946111002	1.582228116
C	0.547401039	-15.004706064	1.338171097
C	-0.777175055	-14.857580078	1.769814127
C	-1.210589087	-13.696241972	2.404796175
C	-2.702993196	-10.769297783	4.626533335
C	-3.509250251	-10.897906799	5.754609433
C	-3.243634234	-11.860101854	6.737845474
C	-2.124927150	-12.684237897	6.556802454

C	-1.297919092	-12.553531911	5.444354400
C	1.000301071	-16.248687157	0.611012045
C	-4.148220296	-12.023024839	7.936383577
H	-4.616965334	-11.073800819	8.217790565
H	-3.598122257	-12.398692901	8.806118612
H	-4.957533355	-12.736818907	7.730805571
H	0.410980030	-17.123632207	0.906009067
H	2.054798147	-16.468933162	0.810933061
H	0.893199062	-16.136057177	-0.476459035
H	2.921081211	1.945570142	0.169925012
H	2.068509151	0.428578031	-0.076344005
H	3.843329278	0.453671033	-0.097290007
H	3.059635220	-1.146626084	3.213000232
H	4.027343290	-1.303020091	1.757860129
H	2.151635155	-2.040040145	0.462179034
H	1.003291070	-1.178169086	1.487539107
H	0.926014068	-6.243906456	0.311948022
H	0.312846022	-8.535382589	-0.048321003
H	-0.369225026	-10.758172784	0.763696054
H	-0.252928018	-9.307857650	4.812700346
H	0.413229030	-7.072511507	3.991217285
H	1.723660123	-11.984892856	2.427440173
H	2.471847177	-14.036341015	1.272499090
H	-1.488313108	-15.662282140	1.596516113
H	-2.242623161	-13.599096988	2.726808194
H	-2.931106210	-10.023173738	3.871822277
H	-4.371007315	-10.243523726	5.865721415
H	-1.886037135	-13.433421964	7.308474500
H	-0.428508031	-13.192984936	5.328087385
H	1.465649103	-4.111917298	0.580529043
H	-2.344276168	3.179283227	2.211698159
H	-1.189481085	3.576531259	0.932492065
H	-2.115843151	4.876425353	1.716743126
H	2.626897190	8.985079637	3.524455254

H	3.486508250	8.794912649	1.990368145
H	4.407001317	9.062472657	3.483593251
H	7.033749486	3.033601218	0.205905015
H	8.249903581	2.147001155	1.151747080
H	8.503378636	3.853955279	0.732999053
C	-1.153837082	4.482586323	4.289784307
H	-1.896485137	3.741444269	4.603466329
H	-1.656876118	5.448588400	4.177179299
H	-0.417766030	4.566197328	5.096853368
C	8.153258564	3.669987264	3.446550246
H	8.652181638	2.710063195	3.622742261
H	7.714515537	4.012653287	4.388802313
H	8.900388629	4.407378319	3.137767224
C	3.615061258	6.804002473	4.832675348
H	3.689289263	5.740324399	5.091079367
H	2.712497193	7.207991520	5.306300363
H	4.485078324	7.302034519	5.270400381

***E*-isomer**

S_1

H	-0.527493000	4.523706000	1.590092000
C	5.093249000	6.839736000	0.881341000
C	6.106985000	5.853439000	0.703797000
C	6.047022000	4.986847000	1.777024000
N	5.017407000	5.429374000	2.588920000
C	4.425702000	6.557283000	2.056446000
C	6.922066000	3.822679000	2.187866000
C	6.057885000	2.626872000	2.521325000
C	6.176195000	1.639712000	3.499126000
C	5.175397000	0.682295000	3.268149000
C	4.441019000	1.088736000	2.138329000
N	4.983450000	2.283387000	1.729198000
C	3.170952000	0.486659000	1.575863000
C	2.233362000	0.864425000	2.697791000

C	1.836899000	0.133397000	3.848242000
C	1.062495000	0.983282000	4.632852000
C	0.982089000	2.233220000	3.981546000
N	1.706137000	2.131894000	2.829403000
C	0.203436000	3.489403000	4.324219000
C	0.670040000	4.619913000	3.430970000
C	0.292019000	4.960596000	2.144085000
C	1.161355000	5.992292000	1.689565000
C	2.059886000	6.269127000	2.701181000
N	1.766698000	5.406291000	3.740355000
C	3.242404000	7.225157000	2.733547000
H	4.884417000	7.667998000	0.219822000
H	6.817782000	5.804713000	-0.108926000
C	7.869584000	3.439964000	1.024679000
H	6.923124000	1.614970000	4.278414000
H	4.979160000	-0.209441000	3.844918000
C	2.766097000	1.109768000	0.228115000
H	2.047425000	-0.921520000	3.995740000
H	0.573975000	0.733333000	5.563139000
C	-1.300983000	3.208224000	4.075814000
H	1.129825000	6.476662000	0.724127000
C	2.874438000	8.516117000	1.975982000
H	4.698967000	2.822956000	0.923324000
H	2.171410000	5.470810000	4.661470000
H	4.688586000	4.935799000	3.405404000
H	1.776895000	2.894058000	2.166557000
C	3.326209000	-1.048921000	1.449049000
C	2.037530000	-1.831372000	1.132498000
C	1.888963000	-3.029100000	2.102291000
N	1.362180000	-4.126097000	1.550886000
N	1.039717000	-5.273020000	2.268524000
C	0.666348000	-6.271031000	1.455770000
O	2.219311000	-2.896112000	3.293578000
C	0.260602000	-7.545303000	1.919493000

C	-0.134843000	-8.572517000	1.006557000
C	-0.537236000	-9.819311000	1.445765000
C	-0.559558000	-10.124182000	2.821590000
C	-0.169726000	-9.131372000	3.740230000
C	0.223545000	-7.876582000	3.314941000
N	-0.970780000	-11.425968000	3.275013000
C	-0.152490000	-12.533827000	2.974506000
C	-2.176821000	-11.544031000	3.993999000
C	1.240151000	-12.371152000	2.853594000
C	2.055313000	-13.449114000	2.523189000
C	1.533217000	-14.730075000	2.300911000
C	0.146815000	-14.884529000	2.418607000
C	-0.685776000	-13.814834000	2.737543000
C	-3.230805000	-10.640942000	3.762622000
C	-4.411968000	-10.724720000	4.493640000
C	-4.605472000	-11.703484000	5.476798000
C	-3.548159000	-12.590904000	5.711446000
C	-2.355193000	-12.515280000	4.997885000
C	2.425613000	-15.885899000	1.912460000
C	-5.907591000	-11.811648000	6.235585000
H	-6.385146000	-10.832116000	6.355996000
H	-5.756079000	-12.234547000	7.235795000
H	-6.628819000	-12.460017000	5.717548000
H	1.996896000	-16.846535000	2.220907000
H	3.417494000	-15.799729000	2.372016000
H	2.578531000	-15.934714000	0.824825000
H	2.628785000	2.197984000	0.269123000
H	1.824758000	0.675240000	-0.115654000
H	3.533651000	0.896828000	-0.522491000
H	3.717769000	-1.444424000	2.388642000
H	4.091631000	-1.229113000	0.686283000
H	2.018480000	-2.141925000	0.081674000
H	1.151809000	-1.199442000	1.276385000
H	0.661791000	-6.120515000	0.364786000

H	-0.120405000	-8.359810000	-0.061443000
H	-0.833468000	-10.586209000	0.734159000
H	-0.191889000	-9.368244000	4.801653000
H	0.519008000	-7.117214000	4.030962000
H	1.669922000	-11.388916000	3.017086000
H	3.129444000	-13.289802000	2.443229000
H	-0.298666000	-15.862399000	2.242809000
H	-1.758010000	-13.967343000	2.802424000
H	-3.103917000	-9.866244000	3.014451000
H	-5.206796000	-10.009152000	4.289551000
H	-3.650646000	-13.351311000	6.484092000
H	-1.547588000	-13.203676000	5.223825000
H	1.156761000	-4.111979000	0.552760000
H	-1.643619000	2.386700000	4.711828000
H	-1.487776000	2.926807000	3.035779000
H	-1.886696000	4.103435000	4.304689000
H	2.020345000	9.002408000	2.456048000
H	2.607690000	8.308884000	0.936016000
H	3.721263000	9.209572000	1.976316000
H	7.307606000	3.176403000	0.123162000
H	8.486627000	2.583382000	1.310294000
H	8.529728000	4.279472000	0.785470000
C	0.408168000	3.821946000	5.821016000
H	0.016437000	3.017910000	6.450517000
H	-0.123840000	4.743817000	6.074433000
H	1.467549000	3.940708000	6.076101000
C	7.771777000	4.213184000	3.420680000
H	8.443604000	3.398206000	3.710524000
H	7.139653000	4.460539000	4.279735000
H	8.378310000	5.090342000	3.179930000
C	3.614700000	7.603666000	4.187906000
H	3.955719000	6.742463000	4.776725000
H	2.759614000	8.055168000	4.703008000
H	4.437392000	8.324301000	4.182600000

***Z*-isomer**

S_0

H	-0.967445069	1.070575079	0.796450056
C	2.652283188	4.932533355	3.814128272
C	4.044142292	4.778720346	3.541453253
C	4.388107318	3.472146247	3.829373276
N	3.239048231	2.845582202	4.266468307
C	2.170012158	3.716984268	4.260160308
C	5.723660396	2.748950198	3.863196280
C	5.503020418	1.280673095	3.569376259
C	5.687376405	0.140287010	4.329388313
C	5.247766380	-0.974950073	3.560485256
C	4.797842346	-0.501400036	2.342971170
N	4.960472358	0.868771060	2.367006173
C	4.175528303	-1.258223089	1.176769084
C	2.878815205	-1.884474136	1.662130118
C	2.563077186	-3.165774227	2.083451148
C	1.226326089	-3.140293226	2.584083184
C	0.753638056	-1.845901131	2.461076176
N	1.766552125	-1.105064079	1.895874138
C	-0.606278046	-1.221383086	2.741082195
C	-0.453831033	0.286127021	2.782651200
C	-0.590784043	1.246111089	1.794684129
C	-0.157809011	2.493409181	2.328786166
C	0.238269017	2.282283165	3.636029261
N	0.063845004	0.936441066	3.886660280
C	0.779047057	3.266428237	4.664796337
H	2.071472147	5.836470402	3.694618263
H	4.719018341	5.549062384	3.194021232
C	6.686655460	3.367443242	2.824126203
H	6.102363448	0.109687008	5.327276367
H	5.235340377	-2.011813143	3.866997276
C	3.907416279	-0.305105022	-0.011964001
H	3.214773230	-4.027123290	2.028122147

H	0.669453046	-3.983049286	2.970633216
C	-1.588661112	-1.615095119	1.611137116
H	-0.136654010	3.444760245	1.814945130
C	-0.160051012	4.490271326	4.726624338
H	4.674542338	1.503920107	1.638181117
H	0.260316019	0.484304035	4.765254341
H	3.195795230	1.855903132	4.466581320
H	1.688151123	-0.111545008	1.729925127
C	5.169587374	-2.367957168	0.733286052
C	4.685529338	-3.221119229	-0.451679032
C	4.922540356	-4.716317341	-0.251667018
N	4.844542349	-5.445248392	-1.432003103
N	5.083799365	-6.823667494	-1.509490110
C	4.351643312	-7.599384560	-0.784871055
O	5.017033362	-5.238418379	0.847178062
C	3.187425228	-7.365267549	0.079929006
C	2.924488208	-8.288608610	1.105161078
C	1.858442135	-8.117511582	1.978311143
C	0.999603070	-7.012632509	1.844318135
C	1.221003089	-6.113010442	0.785885059
C	2.292842167	-6.289749451	-0.073687005
N	-0.035906003	-6.809439505	2.783774199
C	0.230152017	-7.103544525	4.156348300
C	-1.265175091	-6.199759421	2.433664176
C	1.359007097	-6.564499496	4.786667345
C	1.613063115	-6.851417496	6.125426422
C	0.749720054	-7.663634563	6.873831486
C	-0.382883028	-8.183502578	6.232953439
C	-0.639601047	-7.918605549	4.889334351
C	-1.841063134	-6.395375453	1.167878086
C	-3.034214217	-5.762176403	0.829476061
C	-3.714368269	-4.938606357	1.735627123
C	-3.153315225	-4.784402344	3.009146217
C	-1.949479143	-5.391256384	3.356569242

C	1.047310077	-7.989645584	8.318402612
C	-4.980326358	-4.213530304	1.347402097
H	-4.763035342	-3.187430229	1.020298076
H	-5.678400395	-4.143011298	2.189182159
H	-5.496193397	-4.716559339	0.522399038
H	0.131394009	-8.204137581	8.879464643
H	1.564596115	-7.162984518	8.817675632
H	1.694319123	-8.873400642	8.400292580
H	3.228064231	0.514832037	0.251254018
H	3.443154245	-0.844682060	-0.841328060
H	4.844580349	0.135714010	-0.372429027
H	5.357597377	-3.028678218	1.582925116
H	6.124809454	-1.888888136	0.492087035
H	5.104977366	-2.877384210	-1.405140103
H	3.594050260	-3.136261228	-0.543938040
H	4.665326335	-8.642928604	-0.858748062
H	3.579787255	-9.147461645	1.230785090
H	1.688488119	-8.830264653	2.778321202
H	0.580115041	-5.246869377	0.673085049
H	2.450931176	-5.566972388	-0.865000063
H	2.027977148	-5.921463412	4.222577304
H	2.492346181	-6.423680456	6.601698487
H	-1.070283079	-8.815389639	6.790872474
H	-1.518917111	-8.328758611	4.401734315
H	-1.346384098	-7.038568491	0.447823032
H	-3.451327250	-5.923683416	-0.162154012
H	-3.655043261	-4.156793301	3.742337272
H	-1.535107109	-5.241810379	4.347646315
H	5.195900374	-4.978605357	-2.258849164
H	-1.706017121	-2.702107195	1.584096117
H	-1.211165086	-1.291900093	0.635919047
H	-2.571632183	-1.156701084	1.772803126
H	-1.163090085	4.176769303	5.032333363
H	-0.241838018	4.975890360	3.750249270

H	0.216154016	5.226426376	5.445425401
H	6.281708444	3.301247238	1.808637131
H	7.647881538	2.844039206	2.846403205
H	6.865011477	4.425983317	3.042549219
C	-1.156774084	-1.755349125	4.082680292
H	-1.289737091	-2.837825205	4.026831290
H	-2.126649151	-1.298557095	4.309123312
H	-0.471095034	-1.558657111	4.914964353
C	6.346034437	2.902035207	5.270989382
H	7.307290519	2.378819171	5.331593395
H	5.679926383	2.494280181	6.037954428
H	6.506698471	3.961831284	5.490398377
C	0.831709062	2.628586187	6.073948429
H	1.514075108	1.770856130	6.121839447
H	-0.163712012	2.294175164	6.389488462
H	1.193501086	3.364690240	6.797689494

***Z*-isomer**

S_1

H	-0.918292000	0.112930000	1.873845000
C	2.619321000	4.622272000	4.000580000
C	3.989450000	4.545006000	3.616435000
C	4.477214000	3.320901000	4.029502000
N	3.427029000	2.662522000	4.646445000
C	2.287796000	3.442441000	4.636907000
C	5.871558000	2.732325000	4.005979000
C	5.834476000	1.329839000	3.439914000
C	6.586418000	0.192218000	3.731766000
C	6.232999000	-0.806358000	2.809582000
C	5.260171000	-0.272492000	1.945089000
N	5.027281000	1.014995000	2.370012000
C	4.432518000	-0.980409000	0.892508000
C	3.517705000	-1.754860000	1.810757000
C	3.711224000	-3.014194000	2.442552000

C	2.631373000	-3.227483000	3.292605000
C	1.772795000	-2.116758000	3.192284000
N	2.347054000	-1.237624000	2.312071000
C	0.388129000	-1.862357000	3.752000000
C	0.144706000	-0.368424000	3.735257000
C	-0.409663000	0.449933000	2.766496000
C	-0.212343000	1.802006000	3.169604000
C	0.461011000	1.793380000	4.375924000
N	0.693215000	0.468297000	4.690559000
C	0.977472000	2.946270000	5.223056000
H	1.954403000	5.457005000	3.832281000
H	4.558954000	5.316054000	3.117057000
C	6.792067000	3.600509000	3.113345000
H	7.319487000	0.109429000	4.520353000
H	6.615164000	-1.815356000	2.763202000
C	3.670557000	0.003976000	-0.013010000
H	4.508817000	-3.701598000	2.192438000
H	2.428173000	-4.107929000	3.879856000
C	-0.627726000	-2.584196000	2.831919000
H	-0.531269000	2.684040000	2.632492000
C	-0.057667000	4.089028000	5.209705000
H	4.390477000	1.677244000	1.949245000
H	1.061927000	0.144471000	5.571329000
H	3.469525000	1.708018000	4.971630000
H	1.901051000	-0.366020000	2.054011000
C	5.320854000	-1.922515000	0.042116000
C	4.549439000	-2.920112000	-0.838816000
C	4.959927000	-4.384166000	-0.535751000
N	4.584959000	-5.256463000	-1.480553000
N	4.858389000	-6.649941000	-1.456601000
C	3.979010000	-7.363514000	-0.741258000
O	5.501577000	-4.680287000	0.535800000
C	2.771567000	-7.088712000	-0.039141000
C	2.199190000	-8.136406000	0.764500000

C	1.064564000	-7.955987000	1.527651000
C	0.405498000	-6.706801000	1.551141000
C	0.901768000	-5.678785000	0.731068000
C	2.026854000	-5.854239000	-0.050128000
N	-0.717176000	-6.461861000	2.424736000
C	-0.486683000	-6.322063000	3.799976000
C	-1.993040000	-6.325268000	1.819179000
C	0.836486000	-6.220518000	4.287541000
C	1.094121000	-6.087551000	5.650314000
C	0.067656000	-6.043257000	6.601112000
C	-1.240453000	-6.173022000	6.117832000
C	-1.520659000	-6.321751000	4.762302000
C	-2.331898000	-7.151888000	0.733255000
C	-3.549978000	-7.004846000	0.079968000
C	-4.486714000	-6.037867000	0.474112000
C	-4.140226000	-5.210924000	1.546286000
C	-2.917522000	-5.340494000	2.205442000
C	0.355952000	-5.918215000	8.078687000
C	-5.819437000	-5.912767000	-0.225976000
H	-5.709232000	-5.976995000	-1.315195000
H	-6.307155000	-4.959354000	0.005611000
H	-6.508977000	-6.713858000	0.073824000
H	-0.406446000	-5.317448000	8.589153000
H	1.329341000	-5.447416000	8.259122000
H	0.375704000	-6.898825000	8.574233000
H	3.013211000	0.689599000	0.536750000
H	3.047091000	-0.543922000	-0.723374000
H	4.383128000	0.610683000	-0.580259000
H	5.961682000	-2.512753000	0.701483000
H	5.980088000	-1.285943000	-0.558082000
H	4.676219000	-2.690163000	-1.902847000
H	3.470109000	-2.861611000	-0.643744000
H	4.295249000	-8.407715000	-0.700265000
H	2.696603000	-9.104144000	0.778555000

H	0.680983000	-8.766794000	2.142652000
H	0.366710000	-4.732219000	0.702198000
H	2.344740000	-5.046399000	-0.693898000
H	1.660138000	-6.281041000	3.587108000
H	2.130258000	-6.022520000	5.979859000
H	-2.070870000	-6.173285000	6.822081000
H	-2.549342000	-6.454349000	4.450034000
H	-1.617294000	-7.896008000	0.398832000
H	-3.780814000	-7.661334000	-0.756852000
H	-4.833034000	-4.436039000	1.869728000
H	-2.677492000	-4.666403000	3.019400000
H	4.254346000	-4.895232000	-2.367884000
H	-0.443044000	-3.659909000	2.840435000
H	-0.553523000	-2.237101000	1.796987000
H	-1.644204000	-2.394878000	3.188709000
H	-1.004223000	3.740738000	5.632983000
H	-0.251060000	4.443252000	4.193445000
H	0.303978000	4.936261000	5.801264000
H	6.405630000	3.667010000	2.091467000
H	7.795044000	3.165841000	3.076295000
H	6.867119000	4.613778000	3.520601000
C	0.273817000	-2.452041000	5.175874000
H	0.389658000	-3.538194000	5.157773000
H	-0.712845000	-2.223002000	5.588682000
H	1.038829000	-2.051974000	5.852879000
C	6.449780000	2.699594000	5.441355000
H	7.476139000	2.317061000	5.444650000
H	5.845267000	2.068237000	6.100445000
H	6.458801000	3.712807000	5.852386000
C	1.193970000	2.506787000	6.691584000
H	1.967157000	1.734818000	6.794687000
H	0.263971000	2.118154000	7.120768000
H	1.522228000	3.362215000	7.289071000

References

(1) S. H. Vosko, L. Wilk, M. Nusair, *Can. J. Phys.* **1980,** *58*, 1200-1211.

(2) C. Lee, W. Yang, R. G. Parr, *Phys. Rev. B* **1988**, *37*, 785-789.

(3) A. D. Becke, *J. Chem. Phys.* **1993**, *98*, 1372-1377.

(4) A. D. Becke, *Phys. Rev. A: At., Mol., Opt. Phys.* **1988,** *38*, 3098-3100.

(5) M. J. T. Frisch, G. W. Trucks, H. B. Schlegel, G. E. Scuseria, M. A. Robb, J. R. Cheeseman, G. Scalmani, V. Barone, G. A. Petersson, H. Nakatsuji, X. Li, M. Caricato, A. V. Marenich, J. Bloino, R. Janesko, B. Mennucci, H. P. Hratchian, J. V. Ortiz, A. F. Izmaylov, F. Ding, F. Lipparini, J. Goings, B. Peng, A. Petrone, T. Henderson, V. G. Ranasinghe, J. Gao, N. Rega, G. Zheng, W. Ehara, M. Hada, K. Toyota, R. Fukuda, J. Hasegawa, Y. Nakajima, O. Kitao, H. Nakai, T. Vreven, J. A. Throssell, J. E. Peralta Jr., F. Ogliaro, J. J. B. M. Bearpark, E. N. Heyd, K. N. Kudin, V. N. Staroverov, R. Kobayashi, K. Raghavachari, A. Rendell, J. C. Burant, S. S. Iyengar, M. Cossi, J. M. Millam, M. Klene, R. O. C. Adamo, J. W. Cammi, R. L. Martin, K. Morokuma, O. Farkas, J. B. Foresman, D. J. Fox, Gaussian 16, Revision A.03, Gaussian, Inc., Wallingford CT, 2016.

(6) S. Grimme, J. Antony, S. Ehrlich, H. Krieg, *J. Chem. Phys.* **2010**, 132, 154104.

(7) P. C. Harihara, J. A. Pople, *Theor. Chim. Acta* **1973**, *28*, 213-222.

(8) R. Ditchfield, W. J. Hehre, J. A. Pople, *J. Chem. Phys.* **1971**, *54*, 724-728.
